# Atomically engineering interlayer symmetry operations of two-dimensional crystals

**DOI:** 10.1038/s41467-024-55130-z

**Published:** 2024-12-30

**Authors:** Ziyi Han, Shengqiang Wu, Chun Huang, Fengyuan Xuan, Xiaocang Han, Yinfeng Long, Qing Zhang, Junxian Li, Yuan Meng, Lin Wang, Jiahuan Zhou, Wenping Hu, Jingsi Qiao, Dechao Geng, Xiaoxu Zhao

**Affiliations:** 1 School of Materials Science and Engineering, Peking University, Beijing, 100871 China; 2 Key Laboratory of Organic Integrated Circuits, Ministry of Education & Tianjin Key Laboratory of Molecular Optoelectronic Sciences, Department of Chemistry, School of Science, Tianjin University, Tianjin, 300072 China; 3 MIIT Key Laboratory for Low-Dimensional Quantum Structure and Devices & Advanced Research Institute of Multidisciplinary Science, Beijing Institute of Technology, Beijing, 100081 China; 4 Suzhou Laboratory, Suzhou, 215123 China; 5 School of Mechanical Engineering, Shanghai JiaoTong University, Shanghai, 200240 China; 6 Collaborative Innovation Center of Chemical Science and Engineering (Tianjin), Tianjin, 300072 China; 7 Beijing National Laboratory for Molecular Sciences, Beijing, 100190 China; 8 Wangxuan Institute of Computer Technology, Peking University, Beijing, China

**Keywords:** Two-dimensional materials, Structural properties

## Abstract

Crystal symmetry, which governs the local atomic coordination and bonding environment, is one of the paramount constituents that intrinsically dictate materials’ functionalities. However, engineering crystal symmetry is not straightforward due to the isotropically strong covalent/ionic bonds in crystals. Layered two-dimensional materials offer an ideal platform for crystal engineering because of the ease of interlayer symmetry operations. However, controlling the crystal symmetry remains challenging due to the ease of gliding perpendicular to the Z direction. Herein, we proposed a substrate-guided growth mechanism to atomically fabricate AB′-stacked SnSe_2_ superlattices, containing alternating SnSe_2_ slabs with periodic interlayer mirror and gliding symmetry operations, by chemical vapor deposition. Some higher-order phases such as 6 R, 12 R, and 18 C can be accessed, exhibiting modulated nonlinear optical responses suggested by first-principle calculations. Charge transfer from mica substrates stabilizes the high-order SnSe_2_ phases. Our approach shows a promising strategy for realizing topological phases via stackingtronics.

## Abstract (web summary)

van der Waals (vdW) materials offer unique opportunities to engineer their crystal symmetry, but usually require top-down fabrication approaches. Here, the authors report a mica substrate-guided bottom-up method to grow exotic phases of SnSe_2_ and other vdW materials, showing tunable nonlinear optical properties.

## Introduction

The perception of symmetry is paramount in dictating the fundamental regularities of nature, while the diverse textures of the world arise from mechanisms of symmetry breaking^1^. Engineering symmetry is significant to understanding the various guises of nature. In particular, at microscopic scales, various applications of matter in fields such as magnetism, superconductivity, many-body physics, electronics, and optics can be understood and engineered by the preservation or breaking of symmetry^2^. For well-studied topologically protected semimetals, fermionic quasiparticles are directly relevant to the degeneracy of band crossing points near the Fermi level, which is determined by the crystalline symmetry^3^. The Dirac semimetal can transform into a Weyl semimetal when symmetry is broken, due to the splitting of Dirac points^4^. Additionally, symmetry-protected topological phases vanish when the protecting symmetry is broken. A new paradigm of crystal symmetry has recently emerged in the study of materials’ electronic properties^5^. Janus 2D monolayers with broken mirror symmetry, exhibit asymmetric electron distribution that potentially enhances charge density near the Fermi level. These unique 2D manifolds boost their activities in the oxygen reduction reaction^6^ and offer the potential for high-frequency neural stimulation applications^7^. These materials possess low activation barriers and high diffusion coefficients, making them exceptionally suitable for lithium-ion battery anodes. Moreover, the modulation of crystal symmetry has enabled materials to exhibit unique optical responses, expanding their utility in terahertz and mid-infrared detection^8^.

However, current research efforts are predominantly focused on three-dimensional nanoclusters and their assembled heterostructures, with metallic (e.g., Au, Ag, Pt, and Ru) and alloy (e.g., PdAg, PtAg, TaAs, and NbP) nanomaterials considered as exemplary cases^9,10^. The variation in the crystal symmetry is highly correlated to the electronic structures. For example, hcp-Ni crystals (60 µΩ cm) demonstrate significantly higher resistivity compared to fcc-Ni film (6.9 µΩ cm)^11^. The catalytic properties of hcp-Co are superior to ε-Co crystals, primarily owing to the rich electronic density near the Fermi level^11^. So far, there are limited methods to tune crystal symmetries, such as wet-chemical reduction^12^, seed-mediated epitaxial growth^13^, thermal annealing^14^, vapor-liquid-solid^15^, etc.^16,17^. However, the precise design of crystal symmetry, particularly at the atomic scale, targeting metastable or nonstable phases remains challenging.

2D van der Waals (vdW) layered materials belong to a class of highly anisotropic materials, featuring strong intralayer covalent bonds but weak interlayer vdW interaction. The weak vdW coupling offers an additional degree of freedom to modify the crystal symmetry via interlayer gliding and mirror symmetry^18^. This field of research, termed stackingtronics^19,20^, involves interlayer shifts and orientation angles within layers. Stacking engineering has been demonstrated effective in manipulating crystal symmetry, giving rise to correlated unprecedented properties including nonlinear optics (NLO)^21,22^, spin-obit physics^23,24^, piezoelectricity^25,26^, and superconductivity^27,28^. Specifically, AB-stacked bilayer *h*-BN exhibits intriguing out-of-plane ferroelectric polarization in contrast to the conventional AA′-stacked *h*-BN^29^. Similarly, hexagonal MoS_2_ bilayers exhibit sliding ferroelectricity through altering the stacking sequence from centrosymmetric AA′ into AB stacking polytype^30,31^. Transition metal trihalides, like CrI_3_, present stacking registry-dependent magnetism. The rhombohedral ${{\rm{R}}}\bar{3}$ phase taking an ordered ABC stacking order along the armchair direction displays ferromagnetic properties. In contrast, the monoclinic C2/m (ABC stacking along the zigzag direction) is characterized by their antiferromagnetic behavior^32,33^. Analogous transition metal phosphorous trichalcogenides (MPX_3_) are recognized for their electronic and magnetic properties, which vary with stacking configurations (C2/m and ${{\rm{R}}}\bar{3}$ phases), affecting the spin states of transition metal ions and directly determining the exchange energy^34,35^. Moreover, recently discovered layered MnBi_2_Te_4_ has exhibited strong stacking-dependent magnetic properties. Stacking sequences such as AB, AC, AB′, and AA′ predominantly exhibit an antiferromagnetic ground state, whereas configurations like AA and AC′ stackings are predisposed towards a ferromagnetic phase^36^. These phenomena underscore the complexity of interlayer interactions and their impact on material properties.

So far, top-down methods have been widely employed for accurate control over the stacking sequences^37^. However, challenges such as polymer residues, low yield, and interlayer contamination significantly affect the interlayer coupling, thereby hindering applications that require high-quality materials^38^. Conversely, bottom-up methods, e.g., chemical vapor deposition (CVD), offer a superior platform to directly synthesize vdW materials with sharp interfaces, assisted by salt catalysts^39^, thermal energy^40,41^, and the introduction of extra intrinsic atoms^42^. Specifically, sodium chloride is employed as a promoter to reduce the nucleation energy barrier, in which AA-stacking MoS_2_ flakes with a ratio of up to 99.5% have been achieved^39^. A two-step CVD growth process was adopted with reverse gas flow to ensure enough kinetic energy triggering the growth of the bilayer, obtaining AB and AA stacking registries^40^. Furthermore, incorporating Mo as interstitial atoms in the hollow position can induce a transition from AA′ stacking to the thermodynamically unstable AB′ polytype^42^. Certainly, more properties dependent on the stacking polytypes of the aforementioned materials remain undiscovered. Meanwhile, achieving the atomic-scale synthesis of large-area vdW materials featuring higher-order superlattices and controlled interlayer symmetry operations remains a vital challenge.

Herein, we proposed a substrate-guided growth mechanism to synthesize SnSe_2_ with a controlled stacking order down to the single-layer symmetry precision. As demonstrated in T-polymorphic-based SnSe_2_, ordered AA and AB′ stacking registries can be regulated by the charge transfer from the mica substrate and subsequently stabilized by local metal-rich chemical potentials. Highly crystalline SnSe_2_ crystals with distinct stacking polytypes are verified by scanning transmission electron microscopy (STEM) and Raman spectra. The underlying growth mechanism, namely substrate-directed, for synthesizing various configurations has been proposed. The substrate-mediated and controlled chemical potential of the iodide precursor synergistically dictates the SnSe_2_ slabs, i.e., well-ordered interlayer gliding and/or inverted symmetry, leading to the long-range ordering of SnSe_2_ superlattices, e.g., 6 R, 12 R, 18 R, and 18 C. Characterized by periodic inversion symmetry and interlayer gliding operation, higher-order phases exhibit symmetry-dependent NLO responses. This phenomenon, coined as stackingtronics, has also been consistently observed in other T-phase transition metal dichalcogenides (TMDs), such as TiSe₂, providing an alternative approach to modulate crystal symmetry-dependent topological phases in two-dimensional vdW crystals.

## Results and discussion

### Selective growth of AA and AB′-stacked SnSe_2_ crystals

**Fig. 1:**
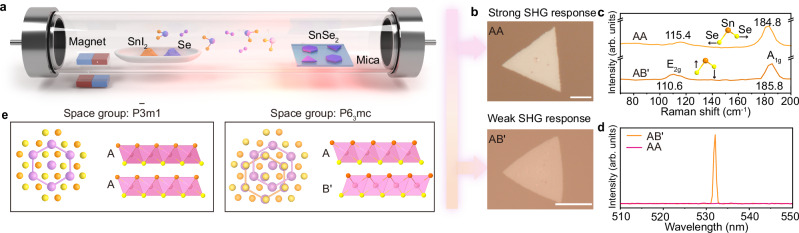
Controlled synthesis of AA- and AB′-stacked SnSe_2_. **a** Schematic illustration of the CVD setup for the phase control in SnSe_2_. Optical images (**b**), Raman spectra (**c**), and SHG (**d**) of AA- and AB′- stacked SnSe_2_ flakes, respectively. **e** Atomic models (top and side views) of AA- and AB′-stacked SnSe_2_. Pink atoms represent Sn atoms, the orange atoms represent the upper-layer Se atoms bonded to Sn, and the yellow spheres represent the lower-layer Se atoms bonded to Sn. Scale bar: **b** 10 μm.

A general ambient pressure CVD route was employed to synthesize SnSe_2_ crystals, as illustrated in Fig. 1a. Selenium (Se) and tin Iodide (SnI_2_) powders were employed as chalcogen and metal precursors, respectively, with mica as the growth substrate. It is known that the sublimation temperature of metal precursors, determining the metal chemical potential, are key parameters to control the phase of 2D materials^43^. Accordingly, the SnI_2_ precursor was chosen for the ease of decomposition at elevated temperatures, benefiting the formation of a more stable and uniform growth environment^44^. Meanwhile, we observed the temperature-dependent crystal shape transformation from sharply defined triangles to rounded edges in SnSe_2_, eventually culminating in fully rounded forms (Supplementary Fig. 1). When the sublimation temperature was set at 320 °C, triangular-shaped single-crystalline SnSe_2_ flakes were obtained. On the other hand, when the growth temperature was elevated to 340 °C reaching a metal-rich condition, the edges become rounded (Fig. 1b and Supplementary Fig. 2), suggesting a nonequilibrium growth condition between dynamics and thermodynamics^45^. When the substrate temperature was further increased to 750 °C, the obtained crystals predominantly consisted of SnSe (Supplementary Fig. 3). The thickness of the as-grown SnSe_2_ flakes typically ranges from bilayer (~1.4 nm) to tens of nanometers through modulating the gas flow of H_2_, as confirmed by atomic force microscopy (AFM) (Supplementary Fig. 4).

The two types of SnSe_2_ flakes grown at different conditions are highly crystalline, as evidenced by X-ray diffraction (XRD), selected area electron diffraction (SAED) (Supplementary Fig. 5), and Raman spectra. XRD measurement exhibits remarkable (00*l*) reflections from both SnSe_2_ and mica substrate, indicating excellent out-of-plane alignment (Supplementary Fig. 6). Characteristic Raman peaks at approximately 115.4 cm^−1^ and 185.2 cm^−1^, corresponding to the *E*_2g_ and *A*_1g_ vibrational modes^46^, respectively, serve as fingerprints to identify SnSe_2_ and confirm the absence of other by-products, e.g., SnSe (Fig. 1d). It is known that Raman spectroscopy is insensitive to stacking sequences^47^. Therefore, second harmonic generation (SHG) measurements are employed to unveil the crystal symmetry of the as-grown SnSe_2_ crystals. Typically, conventional T-phase TMDCs, adopting an AA interlayer stacking registry, belong to ${{\rm{P}}}\bar{3}{{\rm{m1}}}$ space group (Fig. 1c). Consequently, AA-stacked T-phase TMDCs are optically dark for NLO due to their inherent centrosymmetry unless spontaneously broken centrosymmetry through interlayer gliding or inversion symmetry operations. Notably, the crystals grown at 320 °C show no SHG emission, indicating a centrosymmetric AA stacking arrangement. On the other hand, crystals synthesized at 340 °C demonstrate a robust SHG signal implying a unique interlayer stacking configuration with a broken centrosymmetry (Fig. 1e and Supplementary Figs. 7–11).

**Fig. 2:**
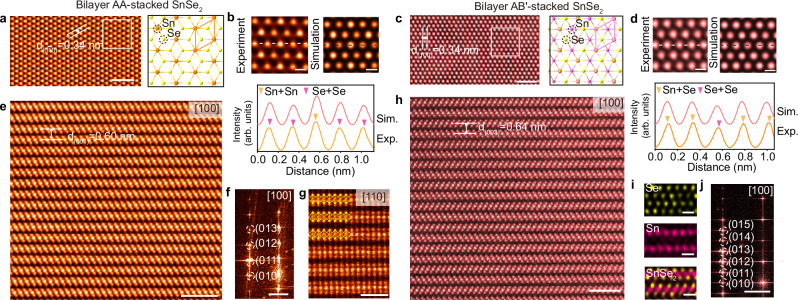
Atomic-resolution images of AA- and AB′-stacked SnSe_2_. **a** Annular dark field scanning transmission electron microscopy (ADF-STEM) image of AA-stacked SnSe_2_ along the [001] direction. The corresponding atomic model was depicted on the right panel. **b** Enlarged ADF image from a with simulated image calculated from AA-stacked SnSe_2_. Intensity line profiles derived from the experiment and simulation were shown in the lower panel. **c** ADF-STEM image of AB′-stacked SnSe_2_ along the [001] direction. The atomic model was depicted on the right panel. **d** Enlarged ADF image from c and corresponding simulated image derived from AB′-stacked SnSe_2_. Intensity line profiles extracted from the experiment and simulation were depicted in the lower panel. **e** Cross-sectional ADF-STEM image of AA-stacked SnSe_2_ along the [100] zone axis and **f** its corresponding fast Fourier transformation (FFT) pattern. **g** Cross-sectional ADF-STEM image of AA-stacked SnSe_2_ along the $[1\bar{1}0]$ zone axis. **h** ADF-STEM image of AB′-stacked SnSe_2_ along the [100] direction. **i** Corresponding EDS mapping showing Se, Sn, and overlaid image. **j** The FFT pattern derived from h. Scale bars: **a**, **c** 1 nm, **b**, **d** 0.2 nm; **e**, **h** 2 nm; **f**, **j** 2 nm^−1^; **g** 1 nm; **i** 0.5 nm.

To atomically elucidate the atomic structure and stacking sequences of two distinct SnSe_2_ crystals, we have carried out annular dark field scanning transmission electron microscopy (ADF-STEM) imaging. It is known that the intensity of the atom blobs in the ADF image is nearly proportional to the atomic number Z^1.6-1.7^, which has been widely employed to atomically visualize the atomic structure and stacking polytypes of 2D materials^48^. The ADF-STEM images (Fig. 2a) of flakes grown at 340 °C reveal that they are highly crystalline and free of topological defects. The projected Z-contrast pattern (Fig. 2b) greatly resembles the AA-stacked SnSe_2_ as corroborated by the image simulation (Fig. 2b and Supplementary Fig. 12a–c), where the two dim atom blobs correspond to the Se (Z = 34) dimer sites, and the bright atom blobs are composed of pure Sn (Z = 50) atoms. On the other hand, the contrast pattern is distinct in crystals grown at 340 °C. Notably, there are two bright atom blobs and one dim atom blob in each unit cell. This configuration occurs due to interlayer sliding, which alters the intrinsic overlap of Se and Se atoms in bilayers being altered to an overlap of Se and Sn atoms, consequently reducing atomic brightness. The contrast disparity between bright and dim atom blobs is largely reduced compared to the ones in AA-stacked SnSe_2_ (Fig. 2c). This unique contrast pattern is due to the interlayer AB′ stacking registry having a *P*6_3_*mc* space group, in which one SnSe_2_ slab undergoes a mirror symmetry operation plus an interlayer gliding along the [120] direction for $\frac{1}{\sqrt{3}}$ a against the other, as further verified by the image simulation (Fig. 2d and Supplementary Fig. 12d–f).

To accurately discern the atomic-scale stacking sequences of SnSe_2_, we conducted cross-sectional ADF-STEM imaging. All samples were carefully prepared and cleaned by the focused ion beam (FIB) with low acceleration voltage to largely dissipate the ion damage. Notably, a perfect AA stacking polytype without any structural distortions along the [100] zone axis was observed (Fig. 2e) agreeing well with the top-view results. The corresponding fast Fourier transformation (FFT) pattern (Fig. 2f) exhibits a single set of spots, indicative of a perfect AA stacking order (Supplementary Fig. 13a, b, f, g). The ADF-STEM images captured along the [1$\bar{1}$0] direction (Fig. 2g) further reveal a well-defined AA stacking sequence. In parallel, an ordered AB′ stacking polytype was verified by cross-section ADF-STEM imaging (Fig. 2h), in which every SnSe_2_ slab alternatively shifts laterally by $\frac{1}{\sqrt{3}}$ *a* unit cell and rotates 180° against the other. Atomic-scale EDS mapping (Fig. 2i) confirmed that the AB′-stacked SnSe_2_ crystals are free of antisite defects or impurities. Because of the long-range ordering (more than 25 nm) in AB′-stacked SnSe_2_ along the [1$\bar{1}$0] direction, we found doubling superspots in the corresponding FFT patterns (Fig. 2j and Supplementary Fig. 13c–e, h–j). To confirm the large-scale consistency of the stacking sequence, we performed low-magnification ADF-STEM imaging along with corresponding FFT pattern analysis. These results clearly demonstrate the successful synthesis of pure AA- and unconventional AB′-stacked SnSe_2_ crystals (Supplementary Fig. 14). Based on the demonstration of AB′-stacked SnSe_2_, we conducted piezoresponse force microscopy (PFM). As illustrated in Supplementary Fig. 15, the observed phase hysteresis and butterfly-shaped amplitude loops are an indication of polarization switching, demonstrating the presence of out-of-plane ferroelectricity in AB′-stacked SnSe_2_ crystals.

### Demonstration of substrate-guided growth mechanism

**Fig. 3:**
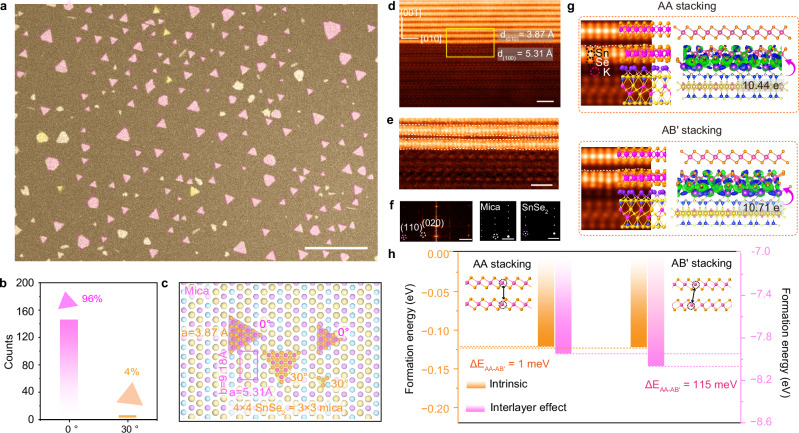
Underlying mechanisms for growing SnSe_2_ with novel phases. **a** Scanning electron microscopy (SEM) image showing epitaxial growth of SnSe_2_ flakes on mica substrate. **b** The statistical counting shows the distribution of 0° and 30° twisted SnSe_2_ flakes grown on mica. **c** Schematic illustration depicting SnSe_2_ flakes grown on mica substrate with different crystallographic orientations. **d** The atomic-resolution cross-sectional ADF-STEM image of AB′-stacked SnSe_2_/mica substrate along the $[1\bar{1}0]$ zone axis. **e** The zoom-in ADF-STEM image reveals atomic distortion as depicted by the white dashed lines along the interface. **f** Corresponding FFT pattern from (**e**). Simulated FFT patterns of mica and AB′-stacking SnSe_2_ were shown on the right panels. **g** The simulated ADF-STEM images derived from the density functional theory (DFT)-optimized AA- and AB′-stacked SnSe_2_ on the mica surface. The right panel showing the differential charge density of AA-, and AB′-stacked SnSe_2_ on mica substrate. The area in green is the region that gained electrons, and the area in blue is the region that lost electrons. **h** DFT calculated formation energies of AA-stacked SnSe_2_ and AB′-stacked SnSe_2_ before and after considering the interface effect, respectively. _Δ_E_AA-AB′_ denotes the difference in formation energy. The orange dashed line represents the formation energy of AA stacking, while the pink dashed line represents the formation energy of AB′ stacking, both represent with and without the influence of the mica substrate, respectively. Scale bars: **a** 10 μm; **b**, **e** 1 nm; **f** 2 nm^−1^.

Next, we atomically unveil the underlying growth mechanism of unique AB′-stacked SnSe_2_. First, we statistically analyze the epitaxial relationship between the SnSe₂ crystal orientation and the mica substrate to examine the substrate effect. After surveying a few hundred flakes, we found that (Fig. 3a) the as-grown triangular SnSe₂ domains predominantly manifest two distinct orientations (0° and 30°) with regards to the mica substrate, corresponding to 96% (0°) and 4% (30°) over the total population, respectively, validating the strong epitaxial relationship between the mica substrate and SnSe_2_ crystals (Fig. 3b, c and Supplementary Figs. 16, 17). To precisely unveil the epitaxial relationship at an atomic level, we performed atomic-resolution ADF-STEM imaging along the interface. Notably, the cross-sectional ADF-STEM images along the [1$\bar{1}$0] direction confirmed an obvious lattice-matching relationship. Both AA- and AB′-stacked SnSe_2_ display the same lattice constant of 3.87 Å, substantially away from the lattice constant (5.31 Å) of the mica substrate (Fig. 3d). The FFT pattern derived from the ADF-STEM image indicates that the (010) crystallographic plane of SnSe_2_ aligns with the (100) crystal plane of mica (Fig. 3e, f). The pattern thus corresponds to a nearly commensurate superlattice comprising 4 × 4 SnSe_2_ unit cells (4 × 3.87 = 15.48 Å) and 3 × 3 mica cells (3 × 5.31 = 15.93 Å). The remaining 2.8% tensile strain could be gradually accommodated by a few SnSe_2_ unit lengths adhered to the mica substrate. Furthermore, we observed a certain degree of ripples at the interface, as labeled by a white dashed line, indicating that interfacial charge transfer is potentially responsible for interlayer distortions (Fig. 3e).

To further elucidate the underlying substrate-guided growth mechanisms, we performed density functional theory (DFT) calculations (Supplementary Note 1). Initially, the formation energy of AA, AB′, AB, and AA′ four stacking registries was assessed (Supplementary Fig. 18 and Supplementary Table 1). Taking AA-stacked SnSe_2_ as the ground state, both AA and AB′ stacking sequences are stable, differing by merely 1 meV/formula unit. Conversely, AA′ and AB polytypes are thermodynamically unstable, exhibiting additional formation energies of 11.5 and 14 meV, respectively (Supplementary Fig. 19) compared to AA stacking registry. Notably, mica substrates, characterized by their layered aluminosilicate structure owning surface potassium ions for charge balance, facilitate charge transfer at interfaces with 2D materials and enhance interfacial interactions (Supplementary Fig. 20). Differential charge density calculations suggest that there is substantial charge transfer along the interface, with 10.71 e^-^ being transferred from the K atoms to bridging Se atoms in AB′-stacked SnSe_2_, and 10.44 e^−^ in AA-stacked SnSe_2_ (Fig. 3g and Supplementary Table 2).

The charge transfer significantly reduces the formation energy of AB′-stacked SnSe_2_ by 115 meV, which has improved the possibility of controlled synthesis for two stacking polytypes (Supplementary Table 3). Calculations indicate that the formation of AB′-stacked SnSe_2_ is attributed to receiving additional electrons supplied by the mica substrates (Supplementary Fig. 21). Thus, the AA and AB′ stacking sequences are believed to be predominantly triggered via the synergistic effect of both temperature thermodynamics and substrate-guided processes (Fig. 3h)^49^. On the contrary, pure AB′ stacking was not observed on sapphire substrates (Supplementary Figs. 22–26). Upon characterizing the stacking behaviors, we found that 75% exhibited BA′ stacking behaviors, 3.6% with AB′ stacking orders, and 21.4% had intrinsic stacking slabs. The weakened charge transfer process between sapphire and SnSe_2_ crystals results in only a 28 meV difference in formation energy difference, highlighting the critical role of the substrate (Supplementary Fig. 27, Supplementary Tables 4, 5, and Supplementary Note 2).

### Minor interlayer gliding in AB’-stacked SnSe_2_ crystals

**Fig. 4:**
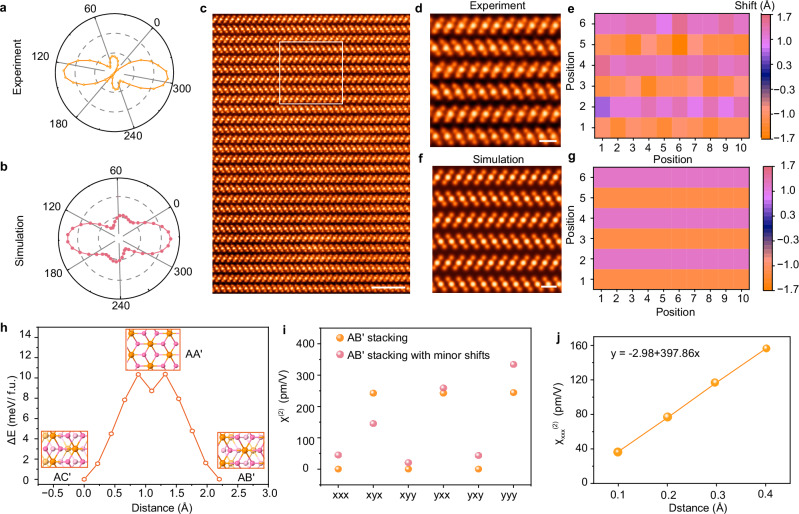
Atomic structure analysis and associated spontaneous SHG. **a** The polarization-resolved SHG response with a 1064 nm excitation laser. **b** Calculated SHG response derived from the AB′ stacking atomic model containing 0.17 Å interlayer gliding along the [120] direction. **c** Cross-sectional ADF-STEM image exhibiting a long-range order AB′-stacked SnSe_2_ along the [100] zone axis. **d** Enlarged ADF-STEM image marked by the white box in (**c**) and corresponding Sn atom displacement map (**e**). **f** Simulated ADF-STEM image of pristine AB′-stacked SnSe_2_ and corresponding Sn atom displacement map (**g**). **h** DFT calculated energy landscape gliding from the AC′ stacking order to the AB′ stacking order using a bilayer SnSe_2_ model along the [120] direction. **i** The nonlinear susceptibility tensor element ${{{\rm{\chi }}}}^{(2)}$ of AB′-stacked SnSe_2_ with minor sliding (orange) and pure AB′ stacking sequence (pink). **j** The ${{{\rm{\chi }}}}_{{{\rm{xxx}}}}^{(2)}$ component as a function of interlayer gliding along the [120] direction. Scale bars: **c** 2 nm; **d**, **f** 0.5 nm.

The as-grown AB′-stacked SnSe_2_ polytype, with a non-centrosymmetric *P*6_3_*mc* space group, exhibits potential for NLO responses. To verify the hypothesis, we conducted linearly polarization-dependent SHG intensity measurements of AB′-stacked SnSe_2_ crystals. Unlike an expected 6-lobe anisotropic SHG polar plot in 3-fold rotational symmetry in AB′-SnSe_2_, we observe an almost azimuthal-polarization dependence of the SHG signal (Fig. 4a and Supplementary Fig. 28). This might be attributed to minor interlayer gliding of SnSe_2_ slabs along the armchair or zigzag direction (Fig. 4b), reducing the intrinsic in-plane sixfold rotational symmetry to a nearly twofold. Therefore, we calculate the polarization-dependent SHG signals by introducing trivial interlayer gliding along the armchair direction for 0.17 Å (Supplementary Fig. 29). To atomically unveil the picometer scale interlayer gliding, we developed customized coding to visualize the degree of the gliding (λ) and successfully detected trivial interlayer sliding (Fig. 4c–g, Supplementary Figs. 30, 31, Supplementary Note 3, and Supplementary Table 6). The minor interlayer gliding is ubiquitous due to the negligible gliding barrier in 2D materials, which can be readily overcome by thermal scattering and other kinetic factors^50^. As suggested by DFT calculation (Fig. 4h and Supplementary Table 7), the gliding barrier is only 1.54 meV/f.u. if the gliding magnitude is less than 0.22 Å along [120] direction in AB′-stacked SnSe_2_. The subtle gliding significantly alters the materials’ optical properties, as evidenced by the observed variation in the frequency-dependent ${{{\rm{\chi }}}}^{(2)}$ (Fig. 4i). Notably, six nonvanishing ${{{\rm{\chi }}}}^{(2)}$ tensors are observed in contrast to AB′-stacked SnSe_2_, which exhibits only three nonzero ${{{\rm{\chi }}}}^{(2)}$ due to the breaking of 3-fold rotational symmetry by interlayer gliding (Fig. 4h and Supplementary Fig. 29b).

We further performed the first-principle calculations on structures with interlayer gliding along the zigzag direction in AB′-stacked SnSe_2_. The resulting SHG polar plots, and the ${{{\rm{\chi }}}}^{(2)}$ components (Supplementary Fig. 32) are robust against the interlayer shift and highly sensitive to local distortion. By adjusting the interlayer gliding to increments as small as 0.1 Å, numerical calculations establish the χ_xxx_^(^²^)^ diagram as a function of shift magnitude, showing a linear dependence of the magnitude of SHG susceptibility |χ_xxx_^(^²^)^| on the gliding distance, which results into a quadratic relationship between SHG signal intensity and shift magnitude (Fig. 4j). To this end, a causal link between interlayer gliding behaviors and NLO response is established. Specifically, a series of NLO responses can be regulated by introducing the interlayer gliding.

### The synthesis of high-order SnSe_2_ stacking polytypes

**Fig. 5:**
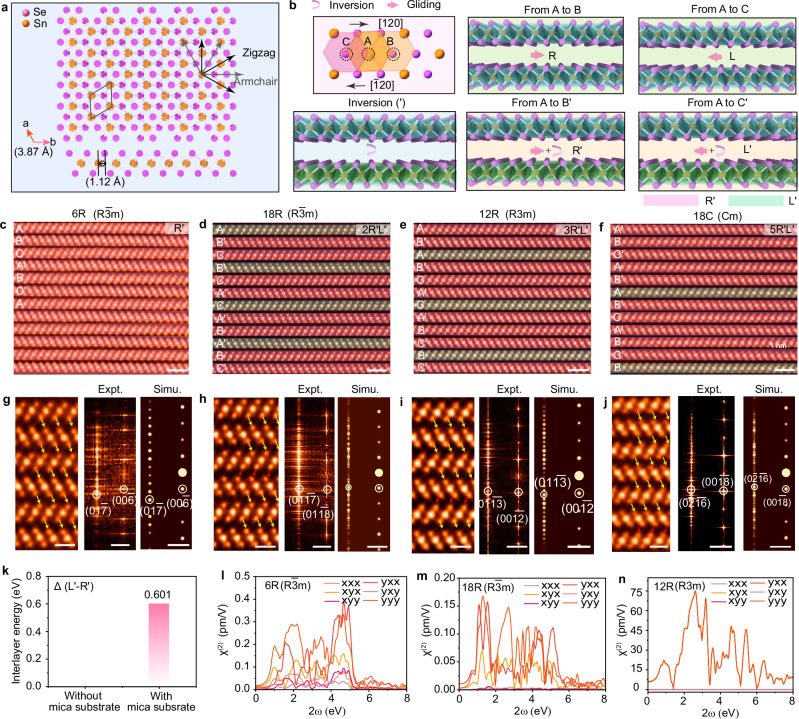
A spectrum of stacking polytypes induced by a variety of interlayer symmetry operations in SnSe_2_. **a** Top and side views of monolayer SnSe_2_. The armchair [120] and zigzag [110] directions are marked with the gray arrow and black arrow, respectively. **b** Atomic models of bilayer SnSe_2_ showing five distinct symmetry operations in sequence, i.e., gliding $\frac{1}{\sqrt{3}}$ a from A to B defined as R, gliding $\frac{1}{\sqrt{3}}$ a from A to C defined as L, interlayer gliding from A to B combining with inversion named as R′, interlayer gliding from A to C combining with inversion behaviors named as L′. Cross-sectional ADF-STEM images of SnSe_2_ superlattices showing 6 R (${{\rm{R}}}\bar{3}{{\rm{m}}}$) (**c**), 18 R (${{\rm{R}}}\bar{3}{{\rm{m}}}$) (**d**), 18 R (*R*3*m*) (**e**), and 18 (*Cm*) (**f**) space groups. The red and yellow false colors were used to label the gliding direction towards the right-hand side direction [120] and left-hand side [$\bar{1}20$], respectively. **g**–**j** Enlarged ADF-STEM images extracted from (**c**–**f**) presenting the unique stacking orders, respectively. Corresponding FFT patterns and simulated FFT patterns were depicted on the right panels. **k** Calculated interlayer energies of R′ and L′ operation symmetry in (**b**). Theoretical calculated ${{{\rm{\chi }}}}^{(2)}$ elements of 6 R (${{\rm{R}}}\bar{3}{{\rm{m}}}$) (**l**), 18 R (${{\rm{R}}}\bar{3}{{\rm{m}}}$) (**m**), 18 R (*R*3*m*) (**n**) stacked SnSe_2_. Scale bars: **c**–**f** 1 nm; **g**–**j** Left, 0.5 nm; Right, 2 nm^−1^.

It has been already demonstrated that one periodic symmetry operation could confer rich NLO responses. Next, we systematically unravel a spectrum of NLO variations upon precisely interlayer symmetry operations in SnSe_2_. It is known that 1T-SnSe_2_ is a threefold symmetry, where the Sn layer is sandwiched between two Se layers with Se-Sn-Se atomic stacking. The crystallographic orientation could be classified into two degenerate directions (Fig. 5a), e.g., zigzag directions are along with the basic vectors [100] and [010] directions, armchair directions along [120] (pink arrows). Three high-symmetry sites are indexed as A (Sn atoms), B (top Se atoms), and C (bottom Se atoms), and the distinct AA, AB, and AC stacking orders can be formed via the gliding of a SnSe_2_ monolayer along the armchair direction for 0, $\frac{1}{\sqrt{3}}$ *a*, and $\frac{2}{\sqrt{3}}$ *a*, respectively (Fig. 5b). The AB′ polytype can be regarded as imposing a periodic in-plane interlayer gliding along the [120] direction for $\frac{1}{\sqrt{3}}$ *a* plus an inversion symmetry operation against the bottom layer, in which we denoted the interlayer symmetry operation as R′. In parallel, there exist R, L, and L′ interlayer symmetry operations as illustrated in Fig. 5b. In our experiments, we found that each SnSe_2_ slabs undergo R′, L′, or their combinational operations, rendering various types of high-order superlattices.

In our experiments, we identified four distinct high-order stacking polytypes: (1) the 6 R (${{\rm{R}}}\bar{3}{{\rm{m}}}$) phase, (2) the 18 R (${{\rm{R}}}\bar{3}{{\rm{m}}}$) stacking, (3) the 12 R (*R*3*m*) stacking sequence, (4) the 18 C (*Cm*) monoclinic phase, which are formed by R′, R′R′L′, R′R′R′L′, and R′R′R′R′R′L′ periodic symmetry operations, respectively, as verified by cross-sectional ADF-STEM images (Fig. 5c–f and Supplementary Fig. 33). The numerical values denote the layers per repeating unit in SnSe_2_, while the letters R and C designate the respective crystal symmetries^51^. The aforementioned AB′ stacking polytype is achieved via periodic R′ and L′ symmetry operations. All SnSe_2_ samples demonstrate that the mirror-symmetric pairs of the tilted SnSe_2_ slabs slide alternatively to the right [120] or left [$\bar{1}$20] orientations, leading to the binding of different unit cells with distinct crystal symmetries (Fig. 5g–j), as highlighted by pink and green boxes. The resulting higher-order symmetries are verified by the appearance of additional diffuse streaks in the corresponding FFT patterns, consisting of the emulated patterns^52^ (Supplementary Fig. 34).

To unveil the underlying growth mechanism of different degrees of SnSe_2_ superlattices, we quantified the interlayer energies of various symmetry operations. It can be seen that the interlayer energies of R′ and L′ operations are observed to be the most thermodynamically favored stacking orders as compared to R and L operations by 14 and 43 meV, respectively (Supplementary Fig. 35). When the mica substrate is taken into account, electron transfer from the mica to the SnSe_2_ crystals disrupts the energy degeneracy, resulting in an interlayer energy difference of 0.601 eV. These results lead to the stabilization of higher-order SnSe_2_ stacking polytypes (Fig. 5k and Supplementary Table. 8). In our experiments, various stacking orders can be effectively synthesized within a narrow sublimation temperature range of 320–340 °C. Calculations imply that CVD-grown SnSe_2_ with diverse layer gliding behaviors are highly challenging owing to these narrowly defined growth conditions. For a comprehensive understanding of the growth dynamics, refer to Supplementary Note 4, and the detailed growth conditions based on modulating the gas flow in a range of 40–80 sccm to grow various stacking orders are listed in Supplementary Table 9. Excitingly, the proposed kinetic-guided strategy can be extended to synthesize other TMDs with distinct crystal symmetry. Supplementary Fig. 36 shows that TiSe_2_ displays an alternative in-plane inversion asymmetry with a stacking sequence of AAC′C′BBC′C′…., significantly distinct from the well-documented AA stacking sequence.

Finally, we systematically discuss the stacking-dependent strength of SHG response. The detailed analysis presented in Supplementary Note 5 and Supplementary Table 10, reveals the variations in SHG responses based on different stacked SnSe_2_. As shown in Fig. 5l, m, the calculated χ^(2)^ of ${{\rm{R}}}\bar{3}{{\rm{m}}}\,$ space group is weak, consistent with the space group featuring a threefold rotoinversion axis. Conversely, the χ^(2)^ tensors of *R*3*m* stacking polytype, which pertains to a non-centrosymmetry system, demonstrated a noticeable enhancement at an incident excitation of 1064 nm (Fig. 5n). The interlayer gliding diversifies the crystal symmetry and stacking sequences, paving the way for effective property modulations, such as ferroelectric^53^, ferromagnetism^32,36,54^ and catalytic^55^. The modulation of centrosymmetry via interlayer gliding presents great possibilities for broadening the scope of two-dimensional ferroelectric materials. Despite the intrinsic neutrality and identical composition in each layer, their asymmetrical charge density distribution, resulting from the interlayer configuration, leads to vertical polarity. These observations are further substantiated by theoretical calculations based on SHG measurement. In addition, it is noteworthy that additional optical anisotropic characterization, such as polarized Raman spectroscopy on AB′-stacked SnSe_2_ crystals, and the results are presented in Supplementary Note 6.

In summary, our proposed substrate-guided growth strategy has been demonstrated to be effective in the synthesis of a wide range of SnSe_2_ superlattices with ${{\rm{P}}}\bar{3}{{\rm{m1}}}$, *P*63*mc*, ${{\rm{R}}}\bar{3}{{\rm{m}}}$, *R*3*m*, *Cm* etc., phases. The interlayer symmetry operations can be well controlled into single-atomic-plane precision. The substrate guides the initial atomic arrangements of crystals and provides the momentum for subsequent SnSe_2_ slabs sliding and/or inversion. According to first-principle calculations, crystal symmetry-dependent NLO responses have been systematically established in an atlas of higher-order SnSe_2_ superlattices. We further demonstrated that such substrate-guided grown superlattices with atomic-controlled interlayer symmetry operations can be also achieved in other 2D materials. This work brings the inspiration for the controllable growth of 2D materials with precise crystal symmetry, paving the way for tunning topological trivial polytypes with single-atomic-scale precision, and shed light on new quantum phenomena triggered by stackingtronics.

## Methods

### Synthesis of various polytypes of SnSe_2_

SnSe_2_ flakes were synthesized on fluorophlogopite mica (KMg_3_AlSi_3_O_10_F_2_) substrate via ambient CVD method. The reaction was conducted in a single-zone furnace equipped with a one-inch quartz chamber. ~20 mg SnI_2_ powder (Alfa Aesar, 99.999%) and ~100 mg Se powder (Alfa Aesar, 99 + %) were loaded in an alumina boat upstream. Freshly cleaved mica (Changchun Fluorphlogopite Mica Company Ltd, 10 × 10 × 0.2 mm) put 15 cm away from reaction precursors. Prior to the reaction, the furnace was washed with 200 sccm high-purity Ar gas for 30 min to remove the residual gas. Then, 20 sccm Ar and 0.8 sccm H_2_ were optimized to create the growth environment. The growth zone was heated to 600 °C (SnI_2_ + 2Se + H_2_ = SnSe_2_ + 2HI) for 20 min. Subsequently, SnI_2_ precursors and Se powers were rapidly pushed into the heating zone and kept for another 5 min. By adjusting various gas flows in a range of 40–80 sccm, we intentionally synthesized various SnSe_2_ stacking sequences.

### Sample characterization and image simulation

Optical images were characterized by a Nikon microscope. AFM (Bruker, Dimension Icon) was employed to measure thicknesses. PFM measurements were conducted on flakes exfoliated onto Au film deposited on Si substrate, using a probe with a ~3 N/m spring constant and a conductive Pt/Ir coating layer. Out-of-plane PFM signal was recorded utilizing a driving frequency of 383 kHz and a drive amplitude of 1500 mV. A bidirectional bias sweep between −8 and 8 V was applied to measure the hysteresis loop. Low-magnification EDS mapping (FEI, Tecnai F20) was used to analyze element compositions. Raman spectra were collected by Witec with an excitation light of ~532 nm. The atomic structures of SnSe_2_ flakes were characterized by a cold-field emission transmission electron microscope (Titan Cubed Themis G2 200) operating at 300 kV. The collection angle of the ADF images ranges from 80 to 200 mrad. Image simulations were performed with the Prismatic package, assuming an aberration-free probe with a probe size of ~1 Å.

## Supplementary information

Supplementary Information

Transparent Peer Review file

## Source data

Source data Fig. 1

Source data Fig. 2

Source data Fig. 3

Source data Fig. 4

Source data Fig. 5

## Data Availability

The Source Data underlying the figures of this study are available with the paper. All raw data generated during the current study are available from the corresponding authors upon request. Source data are provided with this paper.

## References

[1] Gross, D. J. The role of symmetry in fundamental physics. *Proc. Natl Acad. Sci. USA* **93**, 14256–14259 (1996). 11607718 10.1073/pnas.93.25.14256 PMC34470

[2] Fischer, M. H., Sigrist, M., Agterberg, D. F. & Yanase, Y. Superconductivity and local inversion-symmetry breaking. *Annu. Rev. Condens. Matter Phys.* **14**, 153–172 (2023).

[3] Huang, L. et al. Spectroscopic evidence for a type II Weyl semimetallic state in MoTe_2_. *Nat. Mater.* **15**, 1155–1160 (2016). 27400386 10.1038/nmat4685

[4] Wang, L.-X., Li, C.-Z., Yu, D.-P. & Liao, Z.-M. Aharonov–bohm oscillations in Dirac semimetal Cd_3_As_2_ nanowires. *Nat. Commun.* **7**, 10769 (2016). 26902716 10.1038/ncomms10769 PMC4766419

[5] Chaney, G., Ibrahim, A., Ersan, F., Çakır, D. & Ataca, C. Comprehensive study of lithium adsorption and diffusion on janus Mo/WXY (X, Y = S, Se, Te) using first-principles and machine learning approaches. *ACS Appl. Mater. Interfaces* **13**, 36388–36406 (2021). 34304560 10.1021/acsami.1c05508

[6] Tang, B. et al. A Janus dual-atom catalyst for electrocatalytic oxygen reduction and evolution. *Nat. Synth*. **3**, 878–890 (2024).

[7] Han, M. et al. Janus microparticles-based targeted and spatially-controlled piezoelectric neural stimulation via low-intensity focused ultrasound. *Nat. Commun.* **15**, 2013 (2024). 38443369 10.1038/s41467-024-46245-4 PMC10915158

[8] Shi, J. et al. Giant room-temperature nonlinearities in a monolayer Janus topological semiconductor. *Nat. Commun.* **14**, 4953 (2023). 37587120 10.1038/s41467-023-40373-z PMC10432555

[9] Yun, Q. et al. Recent progress on phase engineering of nanomaterials. *Chem. Rev.* **123**, 13489–13692 (2023). 37962496 10.1021/acs.chemrev.3c00459

[10] Fan, Z. et al. Synthesis of 4H/fcc noble multimetallic nanoribbons for electrocatalytic hydrogen evolution reaction. *J. Am. Chem. Soc.* **138**, 1414–1419 (2016). 26752521 10.1021/jacs.5b12715

[11] Cheng, H., Yang, N., Lu, Q., Zhang, Z. & Zhang, H. Syntheses and properties of metal nanomaterials with novel crystal phases. *Adv. Mater.* **30**, 1707189 (2018). 10.1002/adma.201707189 29658155

[12] Li, Z. et al. 1T′-transition metal dichalcogenide monolayers stabilized on 4H-Au nanowires for ultrasensitive SERS detection. *Nat. Mater.* 23, 1355–1362 (2024). 10.1038/s41563-024-01860-w 38589543

[13] Huang, B. et al. Seeded synthesis of hollow PdSn intermetallic nanomaterials for highly efficient electrocatalytic glycerol oxidation. *Adv. Mater.* **35**, 2302233 (2023). 10.1002/adma.202302233 37261943

[14] Han, M. et al. Controllable synthesis and magnetic properties of cubic and hexagonal phase nickel nanocrystals. *Adv. Mater.* **19**, 1096–1100 (2007).

[15] Pham, T. et al. Salt-assisted vapor–liquid–solid growth of 1D van der waals materials. *Adv. Mater*. 36, e2309360 (2024). 10.1002/adma.202309360 38479025

[16] Zheng, H. et al. Observation of transient structural-transformation dynamics in a Cu_2_S nanorod. *Science* **333**, 206–209 (2011). 21737738 10.1126/science.1204713

[17] He, Q. et al. In situ probing molecular intercalation in two-dimensional layered semiconductors. *Nano Lett.* **19**, 6819–6826 (2019). 31498650 10.1021/acs.nanolett.9b01898

[18] Du, L. et al. Engineering symmetry breaking in 2D layered materials. *Nat. Rev. Phys.* **3**, 193–206 (2021).

[19] Cheng, W. N. et al. Engineering charge density waves by stackingtronics in tantalum disulfide. *Nano Lett.* **24**, 6441–6449 (2024). 38757836 10.1021/acs.nanolett.4c01771

[20] Wu, S. et al. Atomically unraveling highly crystalline self-intercalated tantalum sulfide with correlated stacking registry-dependent magnetism. *Nano Lett.* **24**, 378–385 (2024). 38117785 10.1021/acs.nanolett.3c04122

[21] Sun, Z. et al. Giant nonreciprocal second-harmonic generation from antiferromagnetic bilayer CrI_3_. *Nature* **572**, 497–501 (2019). 31367036 10.1038/s41586-019-1445-3

[22] Huang, B. et al. Tuning inelastic light scattering via symmetry control in the two-dimensional magnet CrI_3_. *Nat. Nanotechnol.* **15**, 212–216 (2020). 31907441 10.1038/s41565-019-0598-4

[23] Wu, S. et al. Observation of the quantum spin Hall effect up to 100 kelvin in a monolayer crystal. *Science* **359**, 76–79 (2018). 29302010 10.1126/science.aan6003

[24] Song, P. et al. Coexistence of large conventional and planar spin Hall effect with long spin diffusion length in a low-symmetry semimetal at room temperature. *Nat. Mater.* **19**, 292–298 (2020). 32015531 10.1038/s41563-019-0600-4

[25] Zhu, H. et al. Observation of piezoelectricity in free-standing monolayer MoS_2_. *Nat. Nanotechnol.* **10**, 151–155 (2015). 25531085 10.1038/nnano.2014.309

[26] Wu, W. et al. Piezoelectricity of single-atomic-layer MoS_2_ for energy conversion and piezotronics. *Nature* **514**, 470–474 (2014). 25317560 10.1038/nature13792

[27] Wang, Z. et al. Evidence of high-temperature exciton condensation in two-dimensional atomic double layers. *Nature* **574**, 76–80 (2019). 31578483 10.1038/s41586-019-1591-7

[28] Xi, X. et al. Ising pairing in superconducting NbSe_2_ atomic layers. *Nat. Phys.* **12**, 139–143 (2016).

[29] Yasuda, K., Wang, X., Watanabe, K., Taniguchi, T. & Jarillo-Herrero, P. Stacking-engineered ferroelectricity in bilayer boron nitride. *Science* **372**, 1458–1462 (2021). 10.1126/science.abd3230 34045323

[30] Yang, T. H. et al. Ferroelectric transistors based on shear-transformation-mediated rhombohedral-stacked molybdenum disulfide. *Nat. Electron.* **7**, 29–38 (2024).

[31] Meng, P. et al. Sliding induced multiple polarization states in two-dimensional ferroelectrics. *Nat. Commun.* **13**, 7696 (2022). 36509811 10.1038/s41467-022-35339-6 PMC9744910

[32] Han, X. et al. Atomically unveiling an atlas of polytypes in transition-metal trihalides. *J. Am. Chem. Soc.* **145**, 3624–3635 (2023). 36735914 10.1021/jacs.2c12801

[33] Jiang, P. et al. Stacking tunable interlayer magnetism in bilayer CrI_3_. *Phys. Rev. B* **99**, 144401 (2019).

[34] Zhou, J. et al. Composition and phase engineering of metal chalcogenides and phosphorous chalcogenides. *Nat. Mater.* **22**, 450–458 (2023). 35739274 10.1038/s41563-022-01291-5

[35] Wang, F. et al. New frontiers on van der waals layered metal phosphorous trichalcogenides. *Adv. Funct. Mater.* **28**, 1802151 (2018).

[36] Ren, Y., Ke, S., Lou, W.-K. & Chang, K. Quantum phase transitions driven by sliding in bilayer MnBi_2_Te_4_. *Phys. Rev. B* **106**, 235302 (2022).

[37] Xu, Y. et al. Coexisting ferromagnetic–antiferromagnetic state in twisted bilayer CrI_3_. *Nat. Nanotechnol.* **17**, 143–147 (2022). 34845332 10.1038/s41565-021-01014-y

[38] Huang, Y. et al. Universal mechanical exfoliation of large-area 2D crystals. *Nat. Commun.* **11**, 2453 (2020). 32415180 10.1038/s41467-020-16266-w PMC7228924

[39] Luo, X., Peng, Z., Wang, Z. & Dong, M. Layer-by-layer growth of AA-stacking MoS_2_ for tunable broadband phototransistors. *ACS Appl. Mater. Interfaces* **13**, 59154–59163 (2021). 34856097 10.1021/acsami.1c19906

[40] Zhang, X. et al. Transition metal dichalcogenides bilayer single crystals by reverse-flow chemical vapor epitaxy. *Nat. Commun.* **10**, 598 (2019). 30723204 10.1038/s41467-019-08468-8 PMC6363754

[41] Zhang, X. et al. Controllable epitaxial growth of large-area MoS_2_/WS_2_ vertical heterostructures by confined-space chemical vapor deposition. *Small* **17**, 2007312 (2021). 10.1002/smll.202007312 33733558

[42] Cortés, N., Rosales, L., Orellana, P. A., Ayuela, A. & González, J. W. Stacking change in MoS_2_ bilayers induced by interstitial Mo impurities. *Sci. Rep.* **8**, 2143 (2018). 29391439 10.1038/s41598-018-20289-1 PMC5794788

[43] Zhao, X. et al. Engineering covalently bonded 2D layered materials by self-intercalation. *Nature* **581**, 171–177 (2020). 32405019 10.1038/s41586-020-2241-9

[44] Zhou, X. et al. Ultrathin SnSe_2_ flakes grown by chemical vapor deposition for high-performance photodetectors. *Adv. Mater.* **27**, 8035–8041 (2015). 26541236 10.1002/adma.201503873

[45] Higashitarumizu, N. et al. Purely in-plane ferroelectricity in monolayer SnS at room temperature. *Nat. Commun.* **11**, 2428 (2020). 32415121 10.1038/s41467-020-16291-9 PMC7229038

[46] Fu, J. et al. Controllable synthesis of atomically thin 1T-SnSe_2_ flakes and its linear second harmonic generation with layer thickness. *Adv. Mater. Interfaces* **9**, 2102376 (2022).

[47] Deng, Y. et al. Controlled growth of 3R phase tantalum diselenide and its enhanced superconductivity. *J. Am. Chem. Soc.* **142**, 2948–2955 (2020). 31961673 10.1021/jacs.9b11673

[48] Zhao, X., Ning, S., Fu, W., Pennycook, S. J. & Loh, K. P. Differentiating polymorphs in molybdenum disulfide via electron microscopy. *Adv. Mater.* **30**, 1802397 (2018). 10.1002/adma.201802397 30160317

[49] Wang, J. et al. Dual-coupling-guided epitaxial growth of wafer-scale single-crystal WS_2_ monolayer on vicinal a-plane sapphire. *Nat. Nanotechnol.* **17**, 33–38 (2022). 34782776 10.1038/s41565-021-01004-0

[50] Lin, J. et al. A novel Pd_2_Se_3_ two-dimensional phase driven by interlayer fusion in layered PdSe_2_. *Microsc. Microanal.* **23**, 1700–1701 (2017). 10.1103/PhysRevLett.119.016101 28731752

[51] Li, Y. et al. Microstructure evolution and mechanical properties of magnesium alloys containing long period stacking ordered phase. *Mater. Charact.* **141**, 286–295 (2018).

[52] Sears, J. et al. Stacking disorder in α-RuCl_3_ via x-ray three-dimensional difference pair distribution function analysis. *Phys. Rev. B* **108**, 144419 (2023).

[53] Sui, F. et al. Sliding ferroelectricity in van der Waals layered γ-InSe semiconductor. *Nat. Commun.* **14**, 36 (2023). 36596789 10.1038/s41467-022-35490-0 PMC9810696

[54] Yang, S. et al. Controlling the 2D magnetism of CrBr_3_ by van der Waals stacking engineering. *J. Am. Chem. Soc.* **145**, 28184–28190 (2023). 38096486 10.1021/jacs.3c10777

[55] You, P.-Y. et al. Reversible modulation of interlayer stacking in 2D copper-organic frameworks for tailoring porosity and photocatalytic activity. *Nat. Commun.* **15**, 194 (2024). 38172097 10.1038/s41467-023-44552-w PMC10764794

