# Supplementary material for: Atomically engineering interlayer symmetry operations of two-dimensional crystals
Source: Nat Commun. 2024 Dec 30;15:10835. doi: 10.1038/s41467-024-55130-z (PMC11686199; doi:10.1038/s41467-024-55130-z)
Supplement: Supplementary file 1 — Supplementary Information [file 41467_2024_55130_MOESM1_ESM.pdf]

Supplementary Information for

Atomically Engineering Interlayer Symmetry Operations of Two-Dimensional Crystals

Ziyi Han[1,2,#], Shengqiang Wu[1,#], Chun Huang[3,#], Fengyuan Xuan[4], Xiaoxang Han[1], Yinfeng Long[5], Qing Zhang[2,6,7], Junxian Li[1], Yuan Meng[1], Lin Wang[5], Jiahuan Zhou[8], Wenping Hu[2,6], Jingsi Qiao[3], Dechao Geng[2,6,7,*], Xiaoxu Zhao[1,*]

[1]School of Materials Science and Engineering, Peking University, Beijing 100871, China

[2]Key Laboratory of Organic Integrated Circuits, Ministry of Education & Tianjin Key Laboratory of Molecular Optoelectronic Sciences, Department of Chemistry, School of Science, Tianjin University, Tianjin 300072, China

[3]MIIT Key Laboratory for Low-Dimensional Quantum Structure and Devices & Advanced Research Institute of Multidisciplinary Science, Beijing Institute of Technology, Beijing 100081, China

[4]Suzhou Laboratory, Suzhou, 215123, China

[5]School of Mechanical Engineering, Shanghai JiaoTong University, Shanghai 200240, China

[6]Collaborative Innovation Center of Chemical Science and Engineering (Tianjin), Tianjin 300072, China

[7]Beijing National Laboratory for Molecular Sciences, Beijing 100190, China

[8]Wangxuan Institute of Computer Technology, Peking University, Beijing, China

Z. H., S. W. & C. H. contributed equally to this work

Table of contents

1. Supplementary Fig. 1-36
2. Supplementary Table 1-10
3. Supplementary Note 1-6

Additional references

1. Supplementary Fig. 1-36

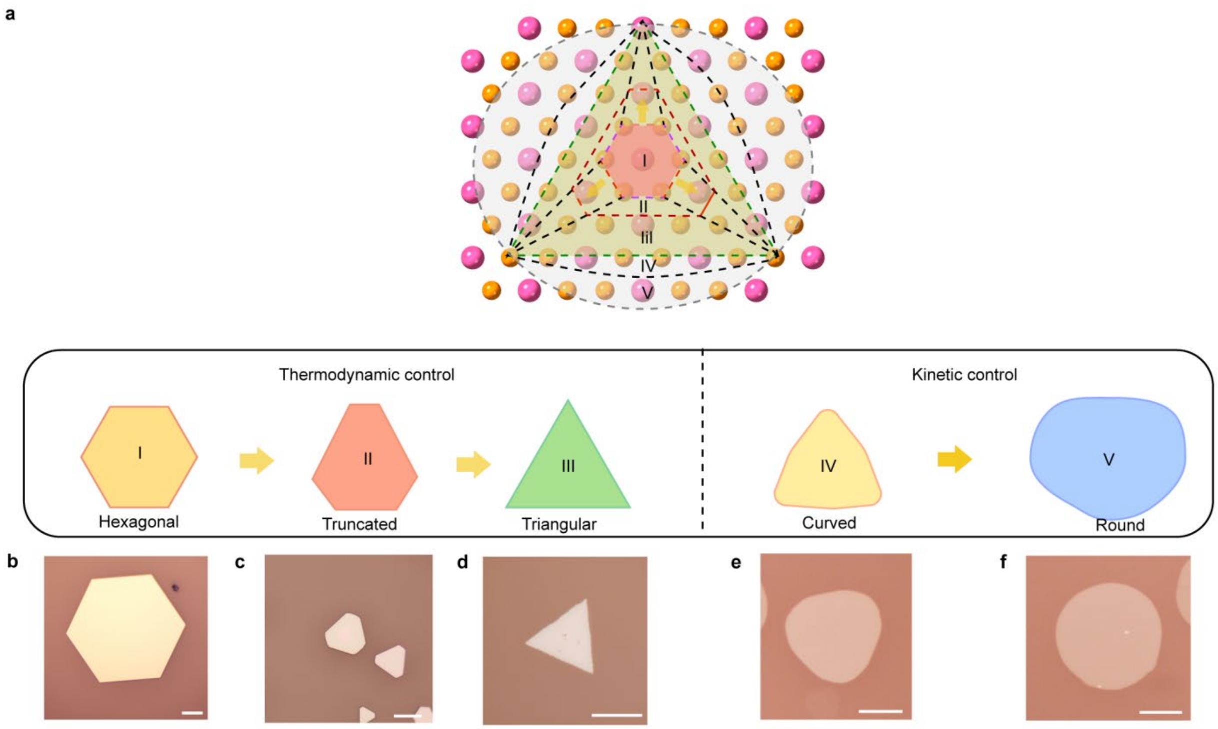

Supplementary Fig. 1. Evolution of $SnSe_2$ shape as a function of growth conditions. (a) Schematic illustration of the morphology evolution of $SnSe_2$ flakes (marked by I, II, III, IV, and V, respectively). The formation of hexagonal, truncated, and triangular $SnSe_2$ flakes is related to thermodynamic control, whereas the formation of curved and round $SnSe_2$ flakes is correlated to kinetic control. Scale bars: 20 µm in (b, e, f); 10 µm in (c, d).

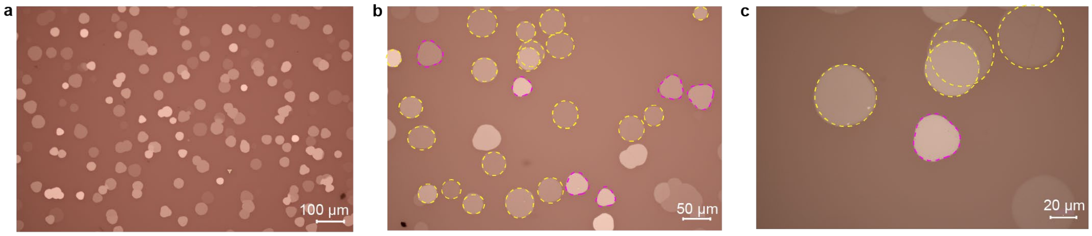

Supplementary Fig. 2. The optical image of AB'-stacked $SnSe_2$ crystals with irregular shapes marked by pink and yellow dashed lines.

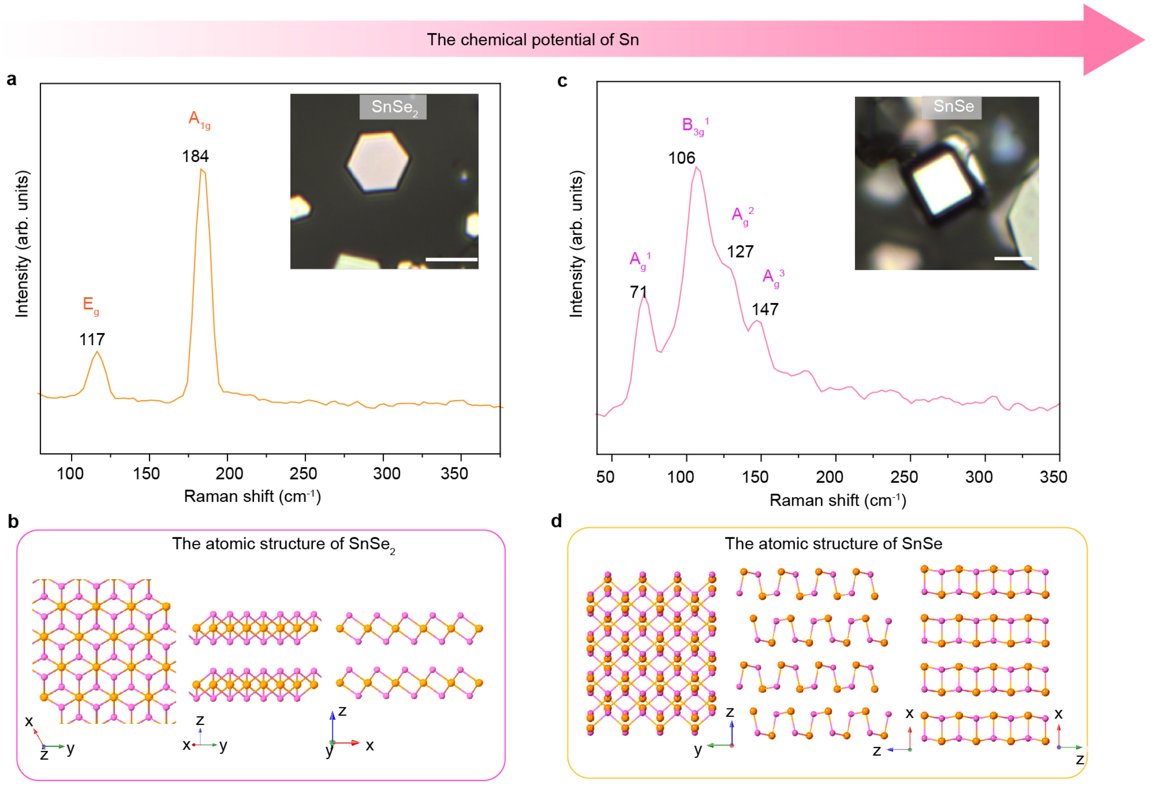

Supplementary Fig. 3. The Raman spectra of $SnSe_2$ and SnSe. The Raman spectra of $SnSe_2$ (a) and SnSe flake (b) and their atomic structure model, respectively. As for $SnSe_2$ crystals, 117 cm^{-1} and 184 cm^{-1} corresponds to the E_{2g} and A_{1g} vibration modes, respectively. As for SnSe, 71 cm^{-1}, 106 cm^{-1}, 127 cm^{-1} and 147 cm^{-1} correspond to the A_g^1, B_{3g}^1, A_g^2 and A_g^3 vibration modes, respectively. Scale bar: 4 µm.

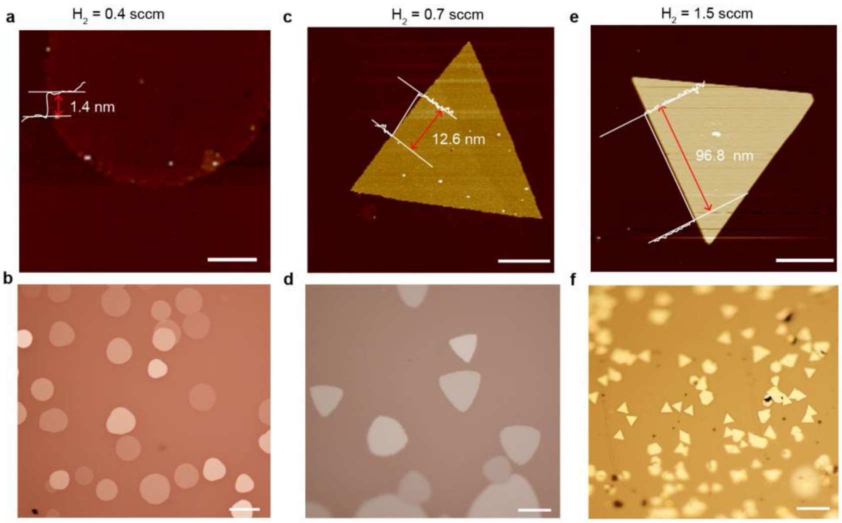

Supplementary Fig. 4. Atomic force microscope (AFM) topological images of $SnSe_2$ with H_2 gas flow at 0.4 sccm, 0.7 sccm and 1.5 sccm. (a, c, e) AFM images of $SnSe_2$ crystals with different thickness. (b, d, f) Optical images of $SnSe_2$ using different gas flow. Scale bars: 4 μm in (a, c, e), 50 μm in (b, f), 10 μm in (d).

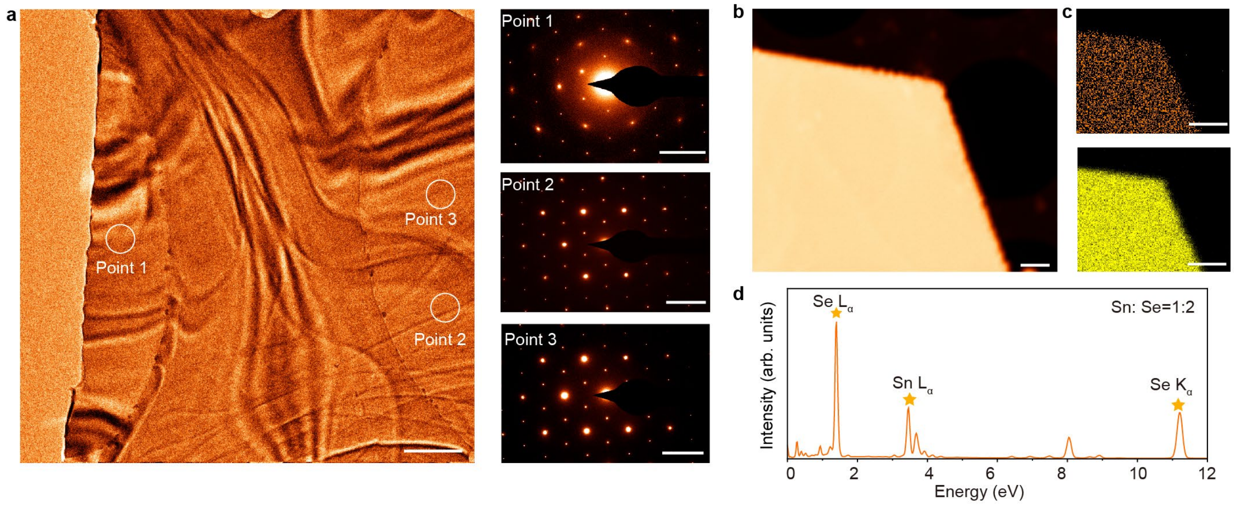

Supplementary Fig. 5. Crystal structures of AA-stacked $SnSe_2$ flakes. (a) Transmission electron microscope (TEM) bright field (BF) image of a typical triangular $SnSe_2$ nanoflake. Corresponding selected area electron diffraction (SAED) patterns taken from the three regions in a were depicted on the right panels (b-d). (e) Low-magnified scanning transmission electron microscopy (STEM) image, corresponding energy dispersive x-ray spectroscopy (EDS) elemental mapping, i.e., (f) Sn and (g) Se, and (h) EDS spectrum of AA-stacked $SnSe_2$. The atomic ratio of Sn and Se is determined as 1: 2. Scale bars: 200 nm in (a); 5 nm^{-1} in SAED patterns (b-d); 1 µm in (e-g).

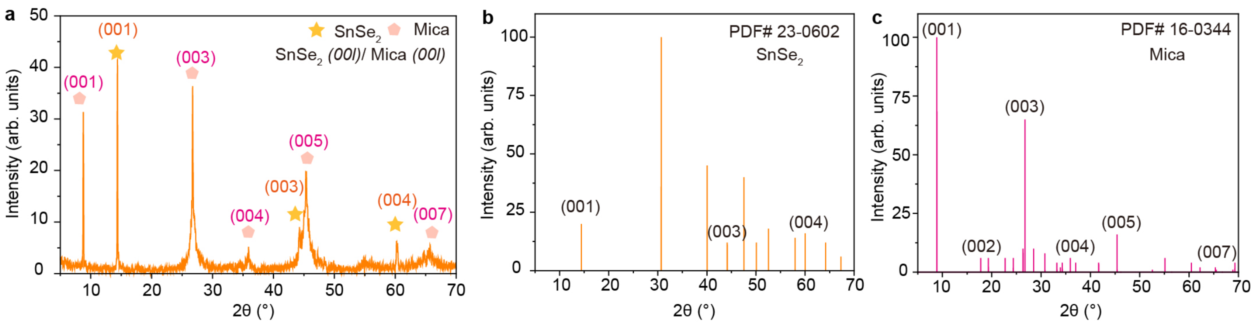

Supplementary Fig. 6. Highly crystalline $SnSe_2$ flakes grown on mica substrates. (a) X-ray diffraction (XRD) pattern of CVD-grown $SnSe_2$ flakes on mica substrates. The standard XRD patterns of (b) AA-stacked T-phase $SnSe_2$ crystal and (c) mica substrate.

Full 2θ scans show the appearance of sharp peaks with the angle of 2θ = 14.36°, 44.31° and 60.22°, which could be referred to as $SnSe_2$ (001), (003) and (004) diffraction peaks, respectively. Based on the (001) peak position, the out-of-plane lattice parameter is calculated to be 6.16 Å, which greatly resembles the lattice arrangement of reference (6.14 Å). All these results show that there is free of strain induced by the lattice mismatch between $SnSe_2$ and mica substrate.

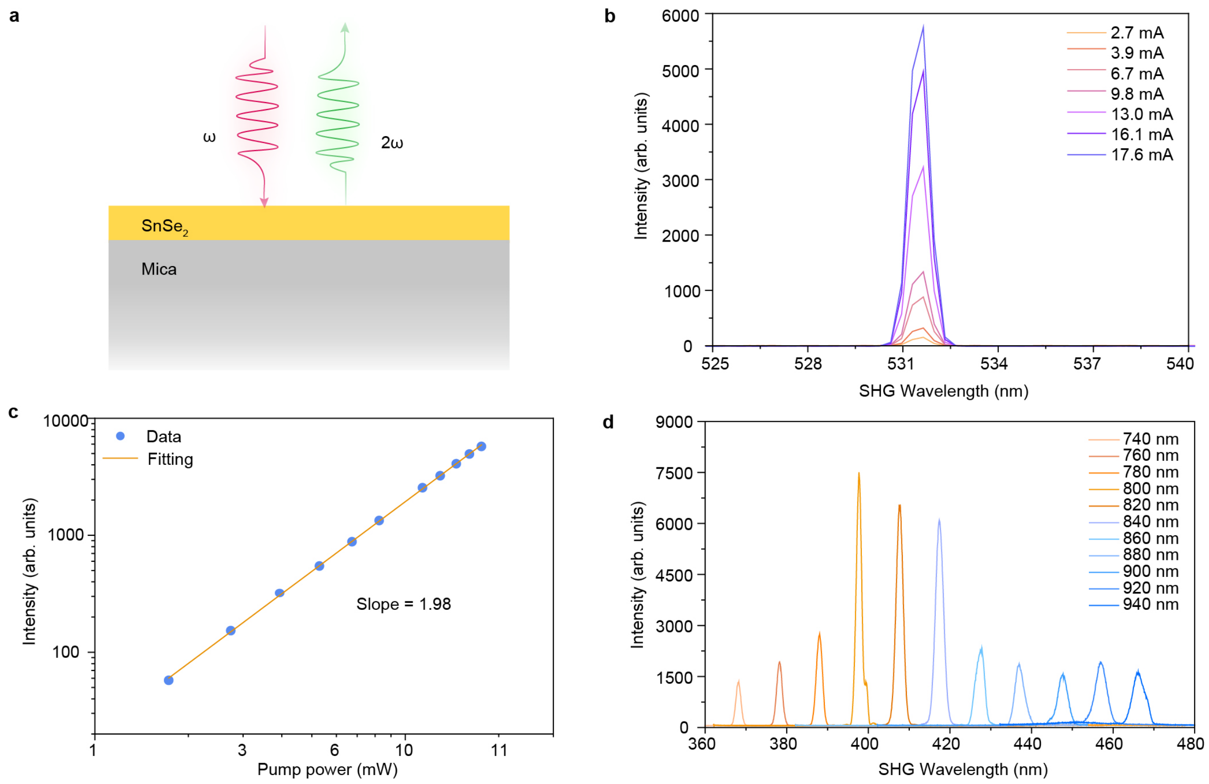

Supplementary Fig. 7. Second harmonic generation (SHG) of $SnSe_2$ (noncentrosymmetry) with an excitation wavelength at 1064 nm. (a) Schematic illustration of SHG process under reflection geometry. (b) Pump power-dependent SHG peak intensity of T-$SnSe_2$, which can be linearly fitted with a slope of ~1.98, confirming a second-order non-linear optics (NLO) process (c). (d) SHG spectra at different excitation wavelengths.

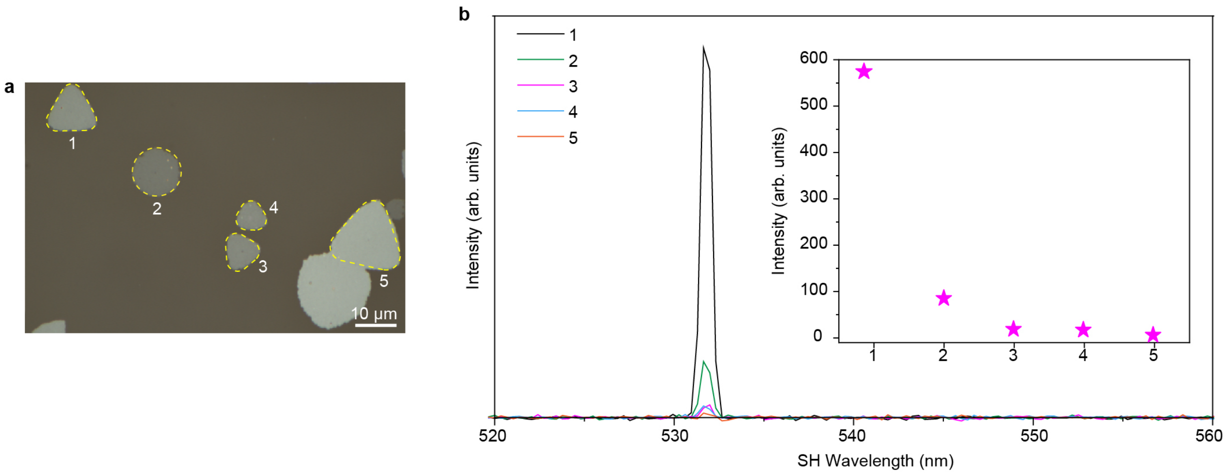

Supplementary Fig. 8. **The SHG characterizations of $SnSe_2$ domains with obvious SHG response.** (a) The optical image of rounded triangle and rounded $SnSe_2$ domains. (b) The SHG intensity of $SnSe_2$ flakes indicated in panel a. Insert image showing the corresponding SHG intensity.

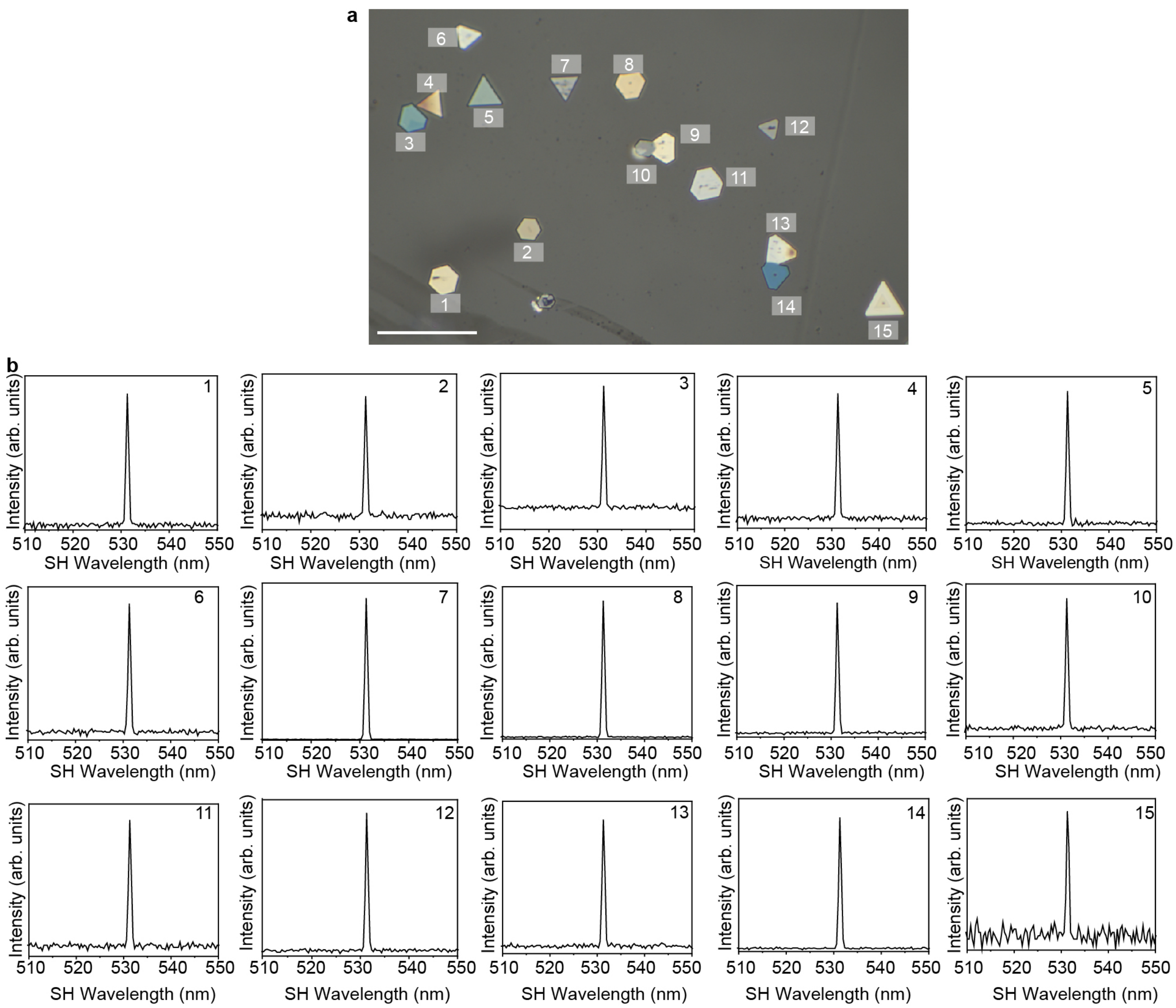

Supplementary Fig. 9. The SHG signals of $SnSe_2$ crystals with broken centrosymmetry on a mica substrate. (a) An optical image of 15 $SnSe_2$ flakes dispersed across the mica substrate. (b) SHG signals obtained from all flakes, with each exhibiting a 100% SHG response. Scale bar: 20 µm in (a).

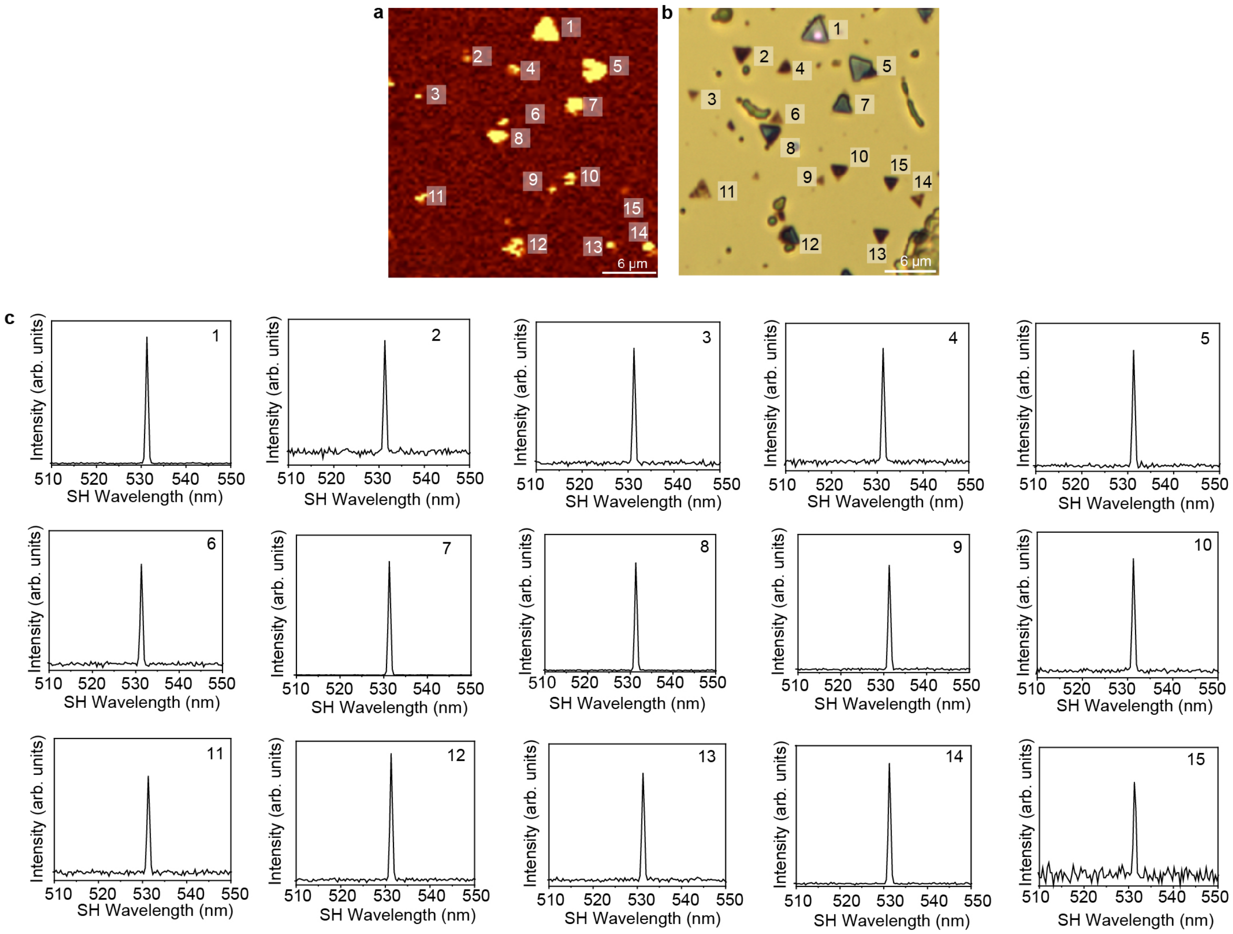

Supplementary Fig. 10. SHG signals of $SnSe_2$ domains transferred onto Au film on Si substrate. (a, b) SHG mapping over a large area with 100% SHG signal coverage. (c) SHG results from 15 individual flakes, demonstrating the controllability of AB'-stacked $SnSe_2$ crystals.

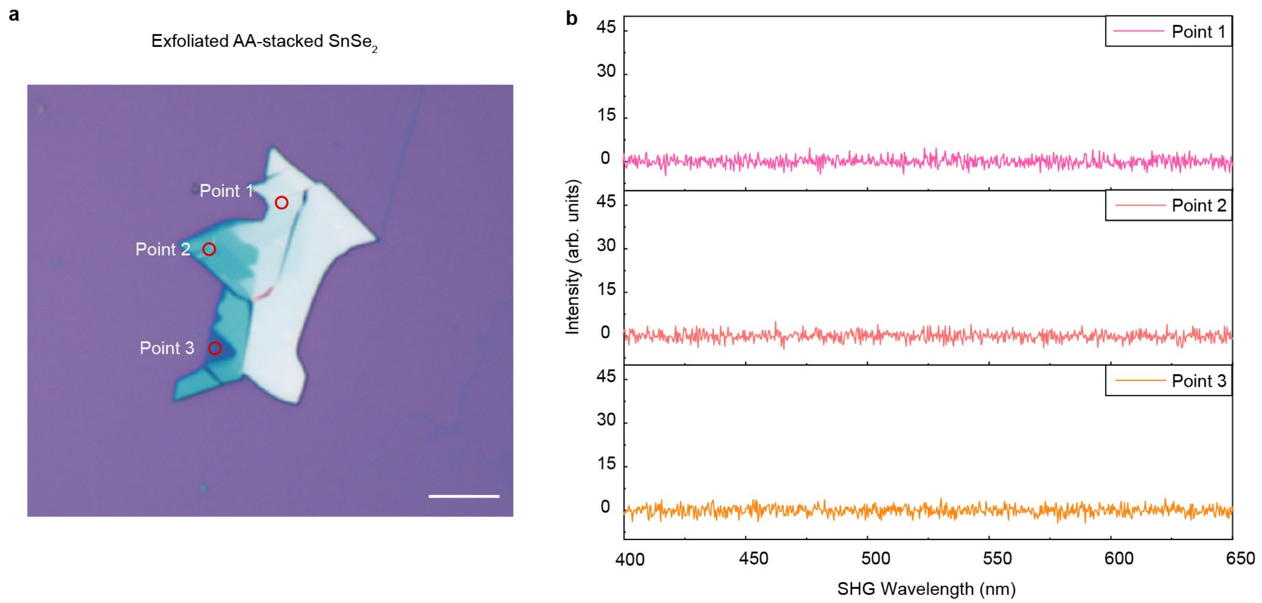

Supplementary Fig. 11. SHG characterizations of AA-stacked $SnSe_2$ on SiO_2 substrate. (a) Optical image of mechanically exfoliated AA-stacked $SnSe_2$. (b) SHG results from the red dot regions in a. There are no SHG emissions for centrosymmetric AA-stacked T-phase $SnSe_2$ crystals (an excitation wavelength at 1064 nm).

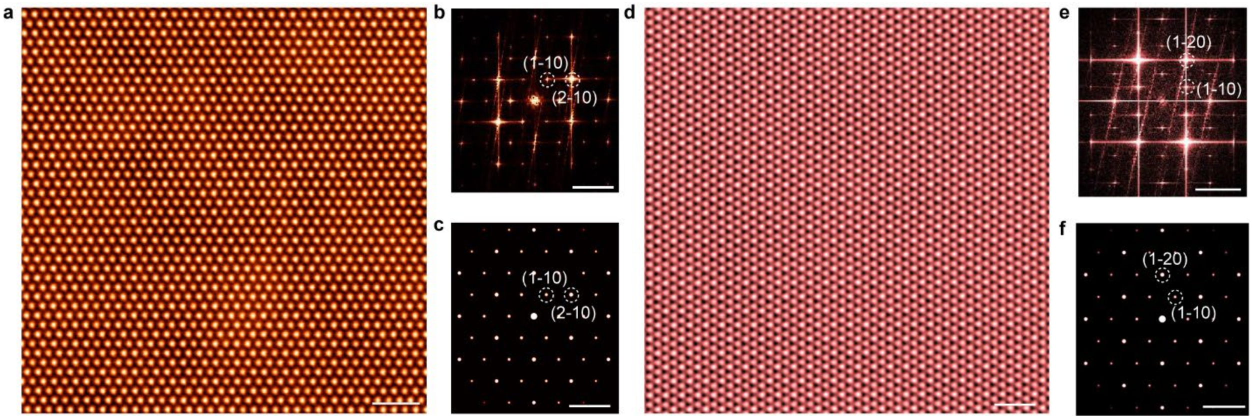

Supplementary Fig. 12. Atomic structure analysis of $SnSe_2$ taking AA and AB' stacking registry. (a) Atomic-resolution annular dark field scanning transmission electron microscopy (ADF-STEM) image of AA-stacked $SnSe_2$. (b) Corresponding fast Fourier transform (FFT) pattern of a and (c) simulated SAED pattern based on an AA-stacked $SnSe_2$ model. (d) Atomic resolution ADF-STEM image of AB'-stacked $SnSe_2$. (e) Corresponding FFT pattern of d and (f) simulated SAED pattern based on AB'-stacked $SnSe_2$. Scale bars: 1 nm in (a) and (e); 5 nm^{-1} in (b-c, f-g).

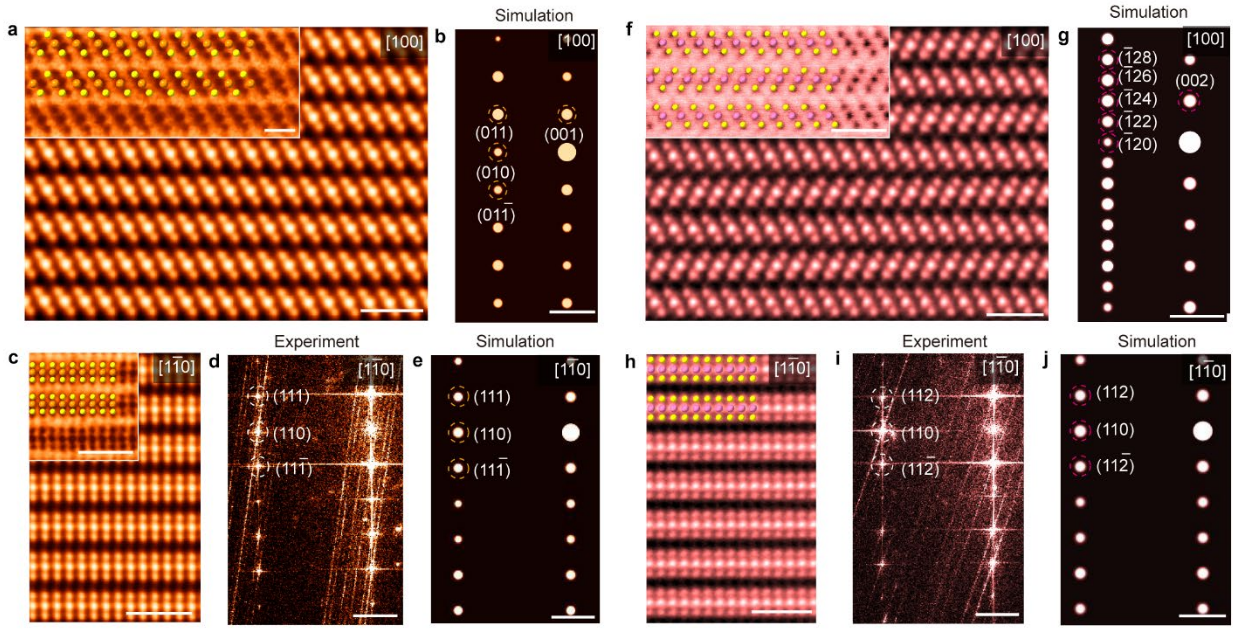

Supplementary Fig. 13. Cross-sectional atomic structure analysis of $SnSe_2$ taking AA and AB' stacking registry. (a) Atomic resolution ADF-STEM images of AA-stacked $SnSe_2$ along the [100] zone axis and (b) simulated SAED pattern based on an AA-stacked model. Annular bright field scanning transmission electron microscopy (ABF-STEM) image taken simultaneously together with the atomic model were overlaid in a. (c) Enlarged ADF-STEM image showing a well-defined AA stacking order along the [1$\bar{1}$0] zone axis. (d) Corresponding FFT pattern from a and (e) simulated SAED at the same region. (f) Atomic resolution ADF-STEM image showing AB'-stacked $SnSe_2$ along the [1$\bar{1}$0] zone axis and (g) simulated SAED pattern. ABF-STEM image taken simultaneously together with the atomic model were overlaid in **f**. (h) Enlarged ADF-STEM image showing a well-defined AA stacking order along the [1$\bar{1}$0] zone axis. (i) Corresponding FFT pattern from **f** and (j) simulated SAED at the same region. Scale bars: 1 nm in (a, c, f, h), 1 nm; 2 nm^{-1} in (b, d, e, g, i, j).

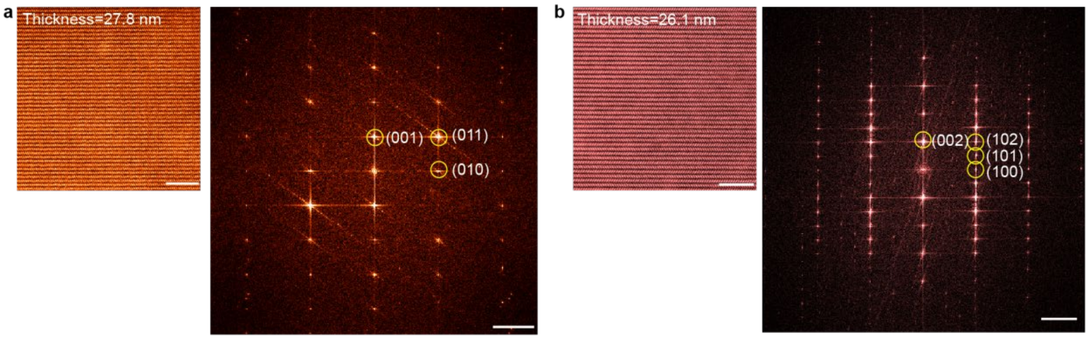

Supplementary Fig. 14. The ADF-STEM images and the corresponding FFT pattern over a large area. (a) The ADF-STEM image of AA-stacked $SnSe_2$ with a thickness of 27.8 nm (left panel) and the corresponding FFT pattern (right panel). The FFT results are in accord with the simulated SAED pattern. (b) The ADF-STEM image of AB'-stacked $SnSe_2$ with a thickness of 21.6 nm and the relative FFT pattern. Analogously, the FFT results are also consistent with simulated SAED results. Scale bars: 5 nm (left panel) in (a, b); 2 nm^{-1} (right panel).

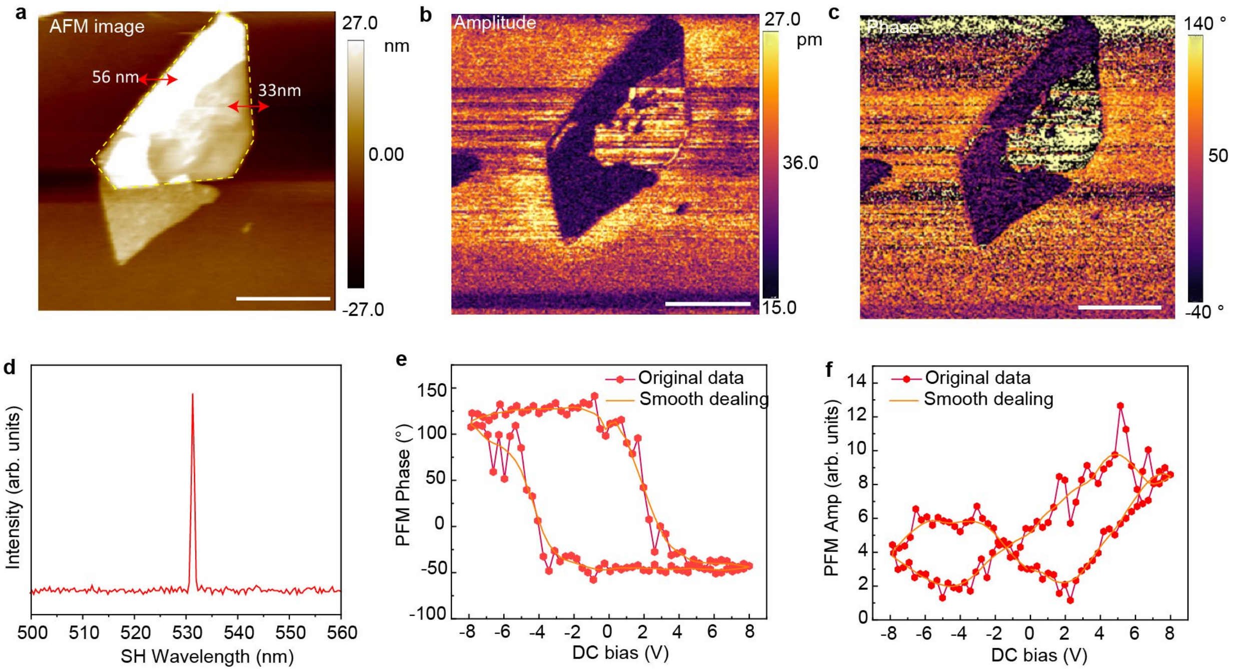

Supplementary Fig. 15. **Piezoresponse force microscopy (PFM) characterizations of AB'-stacked $SnSe_2$ crystals.** The AFM image (a), amplitude image (b) and phase image (c) of the corresponding domain (labelled as yellow dashed line). (d) The domains in a with a significant SHG response. Relative PFM phase hysteretic (e) and butterfly loop (f) of nanosheet with ferroelectric properties. Scale bars: 2 μm in (a-c).

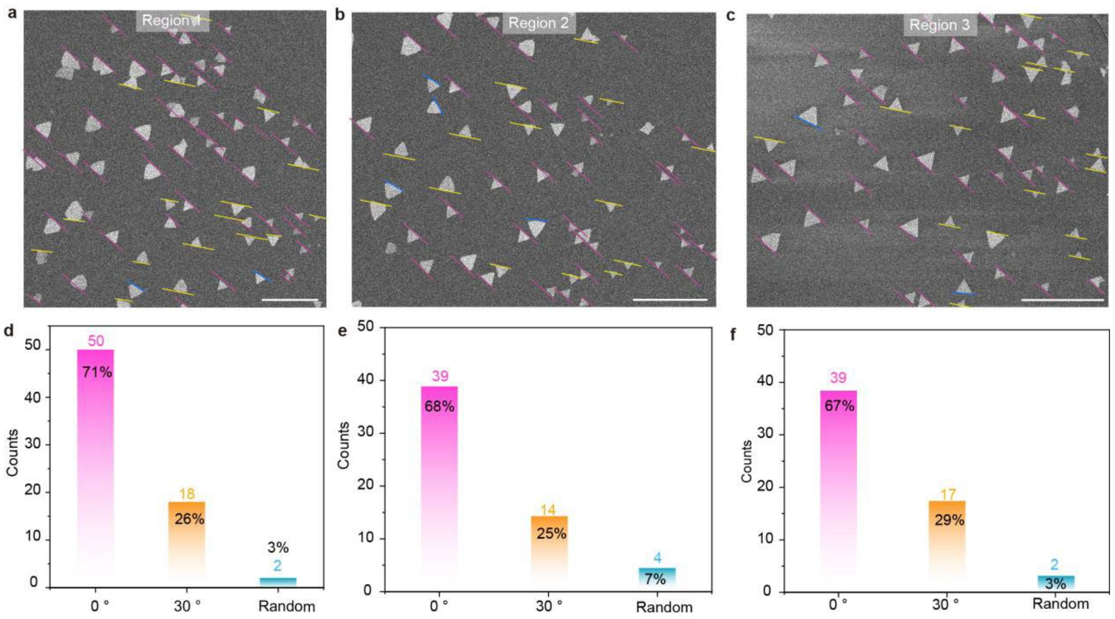

Supplementary Fig. 16. Scanning electron microscopy (SEM) images showing $SnSe_2$ crystals with well-defined epitaxial growth relationship (H_2 = 0.5 sccm, Ar = 20 sccm). (a-c) The orientation of $SnSe_2$ flakes with 0 ° and 30 °, respectively. (d-f) The statistical results of flakes orientations. Scale bars: 10 μm in (a-c).

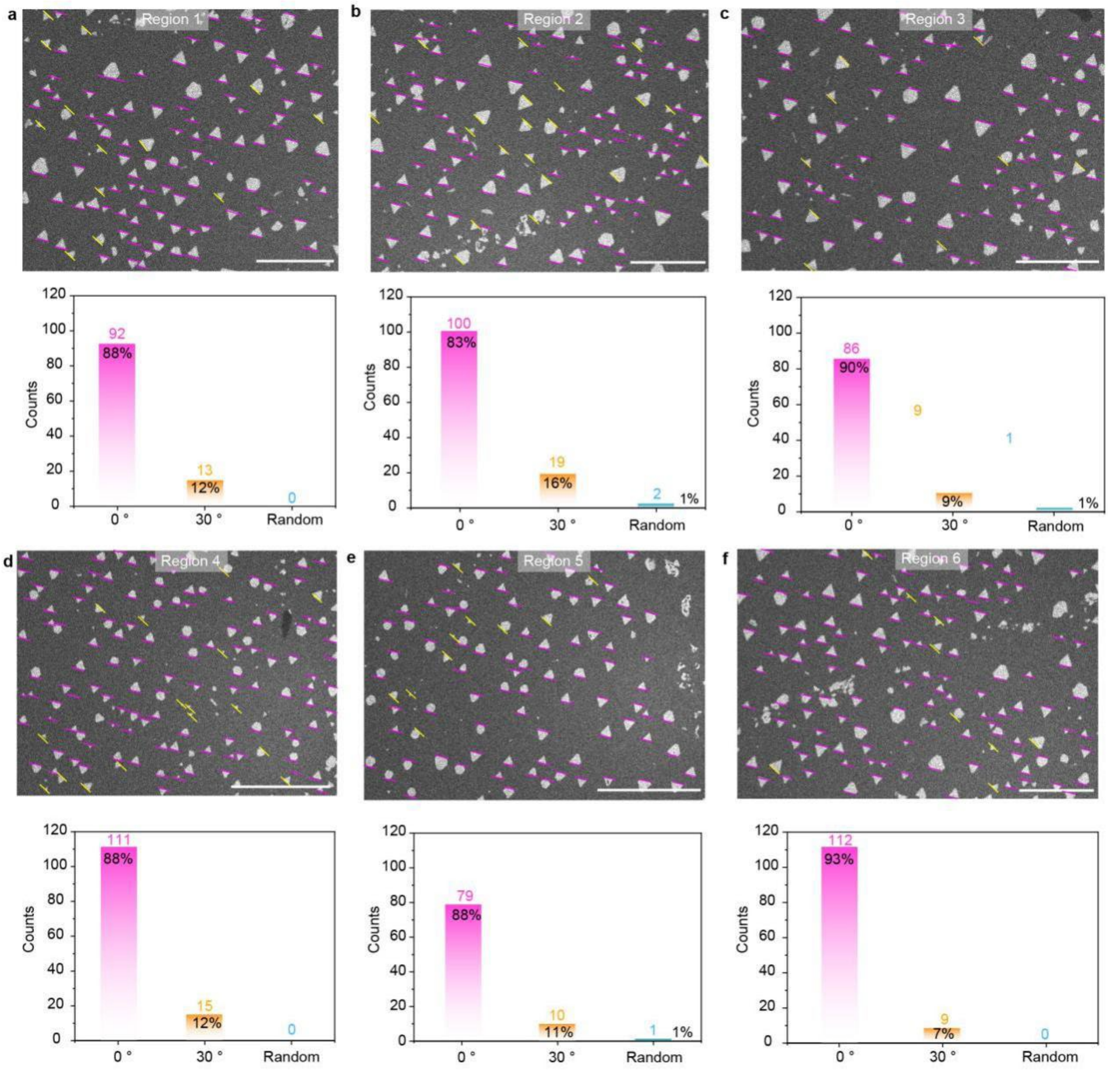

Supplementary Fig. 17. SEM images of $SnSe_2$ crystals grown at 600 °C (H_2 = 1 sccm, Ar = 30 sccm). Six regions are selected to count the amount of aligned $SnSe_2$ flakes. The alignment directions are labelled as 0 ° (pink line) and 30 ° (yellow line), respectively. Scale bars: 50 µm in (a-c, f), 100 µm in (d, e).

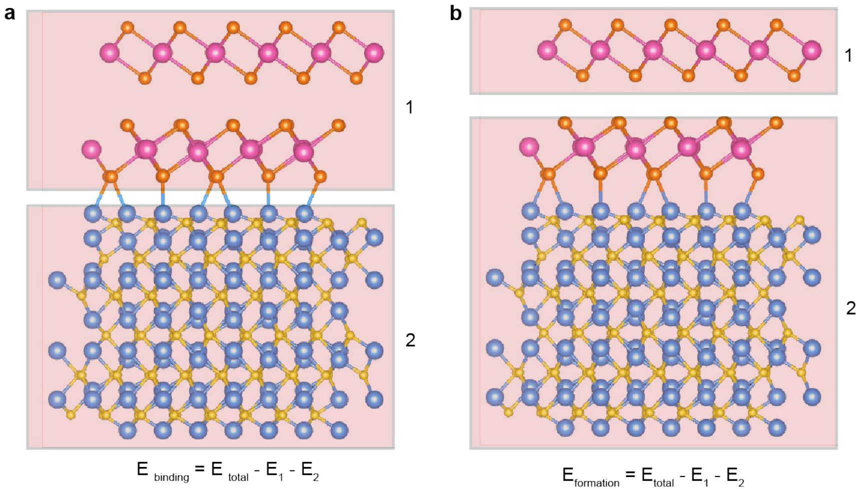

Supplementary Fig. 18. **The definition of binging energy and formation energy.** (a) The interlayer energy meaning the energy difference between the total energy of the supercell containing AA-stacked $SnSe_2$ crystals (Region 1) and the sapphire substrate (Region 2). (b) schematic diagram showing the definition of formation energy.

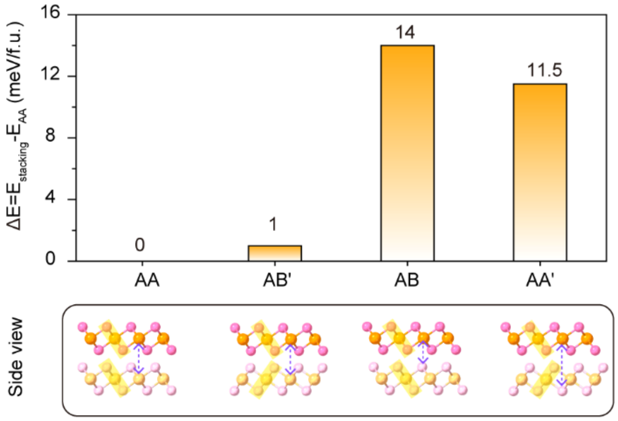

Supplementary Fig. 19. Density functional theory (DFT) calculated the formation energy of various $SnSe_2$ as a function of stacking registry (AA, AB', AB, and AA'). The formation energy of the AA stacking order was set as 0 as the reference.

The binding of $SnSe_2$ crystals with various stacking registries through modulating the growth temperature could be ascribed to a thermodynamically stable state. To verify the hypothesis, we performed the DFT calculation to evaluate the stability. The results consistently show that the stacking energy difference is 1 meV/formula unit, which can be considered as the uncertainty of DFT calculations. However, the formation energy of AA'- and AB-stacked $SnSe_2$ indicates their thermodynamically unstable state, which has therefore not been observed in experimental studies.

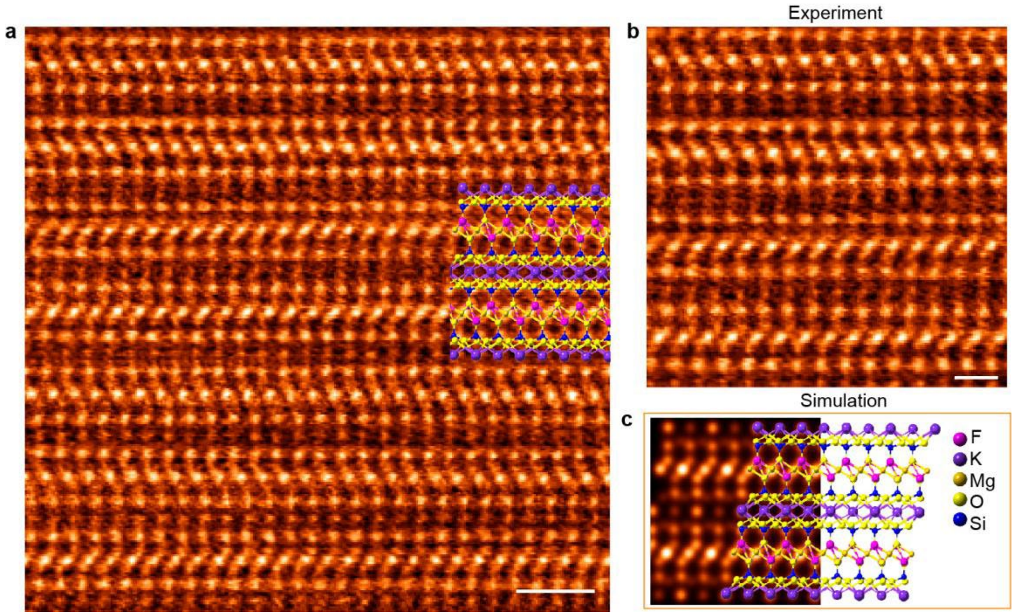

Supplementary Fig. 20. The atomic structure of mica substrate. (a, b) The ADF-STEM image of mica substrate. Each sheet consists of two tetrahedral silicate layers sandwiching an octahedral sheet containing aluminium or magnesium with the layers held together by positively charged potassium. (c) The simulated ADF-STEM image based on mica atomic model. Scale bars: 1 nm in (a), 0.5 nm in (b).

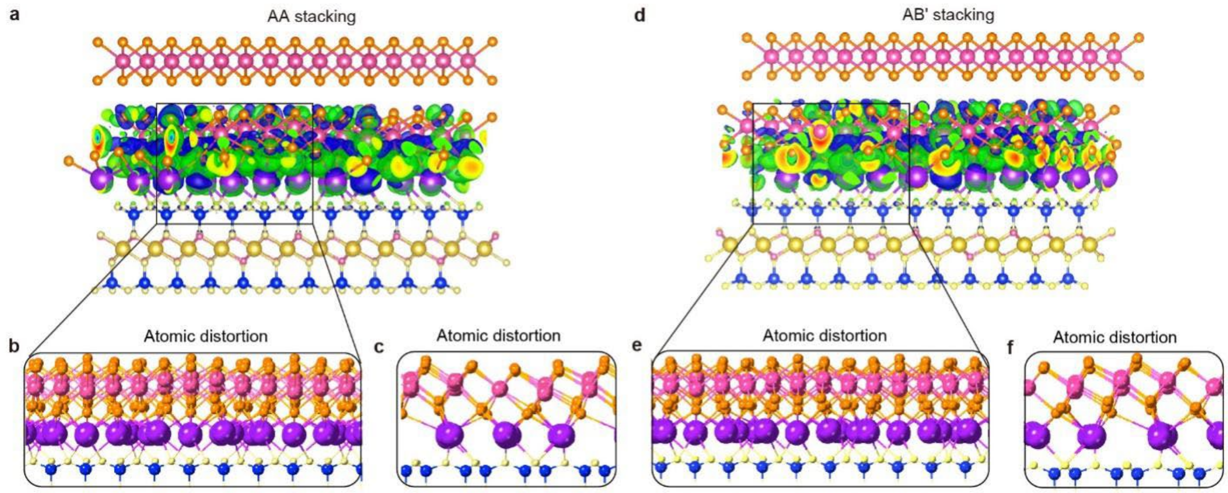

Supplementary Fig. 21. Charge transfer process between $SnSe_2$ with AA and AB' stacking registries. (a) The differential charge density of AA-stacked $SnSe_2$ on mica substrate along the $[1\bar{1}0]$ zone axis. Enlarged atomic model of **a** zooming at the interface and along the (b) $[1\bar{1}0]$ and (c) [100] zone axis. (c) Atomic model of **a** zooming at the interface along the [100] zone axis. (d) The differential charge density of AB'-stacked $SnSe_2$ on mica substrate along the $[1\bar{1}0]$ zone axis. (e, f) Enlarged atomic model of **d** zooming at the interface and along the (e) $[1\bar{1}0]$ and (f) [100] zone axis. Notably, significant structural distortions can be visualized in the zoom-in atomic models, confirming the strong interlayer charge transfer between $SnSe_2$ and the mica substrate. As illustrated in Fig 3g, there are 10.44 e^- and 10.71 e^- transferred from mica substrate to $SnSe_2$ with AA and AB' stacking sequences, respectively. Therefore, the AB' stacking polytype is more thermodynamically stable compared with AA-stacked $SnSe_2$.

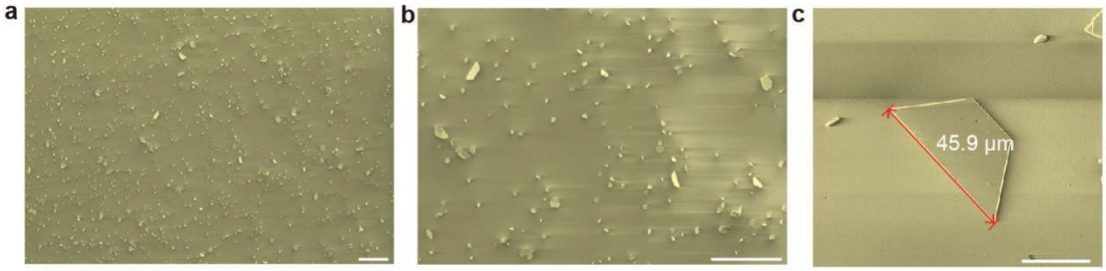

Supplementary Fig. 22. **SEM images of $SnSe_2$ crystal on sapphire substrate.** The crystals grown on the sapphire substrate were free of a significant epitaxial relationship with the underlying substrate. Scale bars: 200 µm in (a, b); 20 µm in (c).

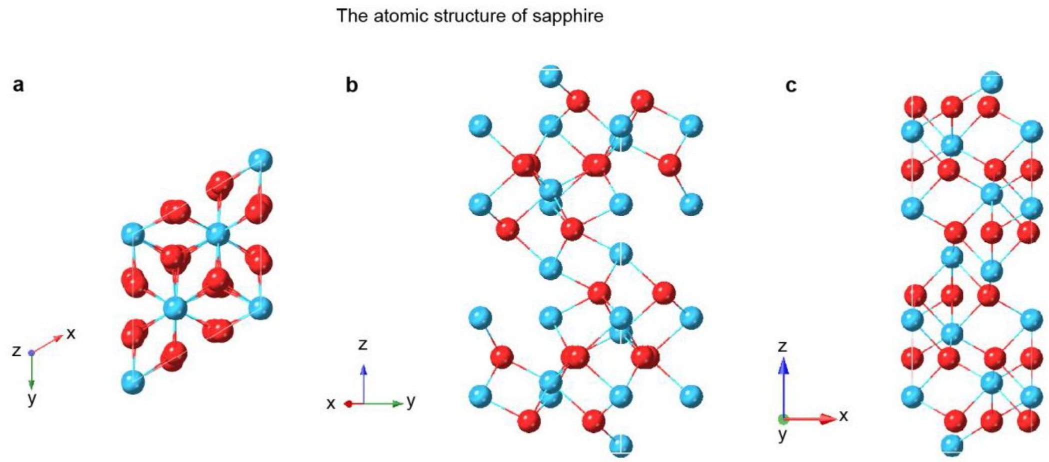

Supplementary Fig. 23. The atomic structure of sapphire with lattice constant a = b = 4.8050 Å, c = 13.1163 Å, α = β = 90°, γ = 120°. The red and blue ball corresponds to Al and O atoms, respectively. The Al cations are arranged in a tightly packed lattice with O anions occupying the spaces between them, creating a repeating pattern of alternating layers of Al and O atoms throughout the crystal.

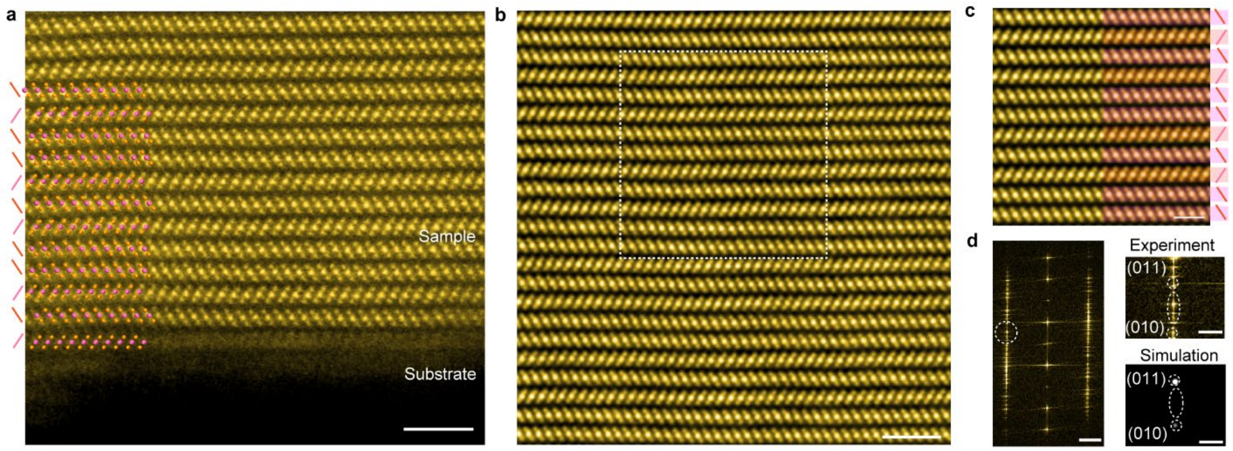

Supplementary Fig. 24. Atomic structure of $SnSe_2$ flakes grown on sapphire substrate. (a) Cross-sectional ADF-STEM images of $SnSe_2$ revealing the mixture of AA, AB', and AC' stacking orders. No well-ordered epitaxial relationship was found between the $SnSe_2$ crystal and the sapphire substrate. (b) The atomic structure of the inner region of as-grown $SnSe_2$ crystal. ADF-STEM image showing the mixed stacking structure with long periodicity. (c) The zoom-in image from the white box region in **b**. (d) The corresponding FFT patterns from b revealing characteristic superspots due to the coexistence of multiple stacking orders. Enlarged FFT patterns together with the simulated pattern were depicted on the right panels. Scale bars: 2 nm in (a); 1 nm in (b, c); 2 nm^{-1} in (d).

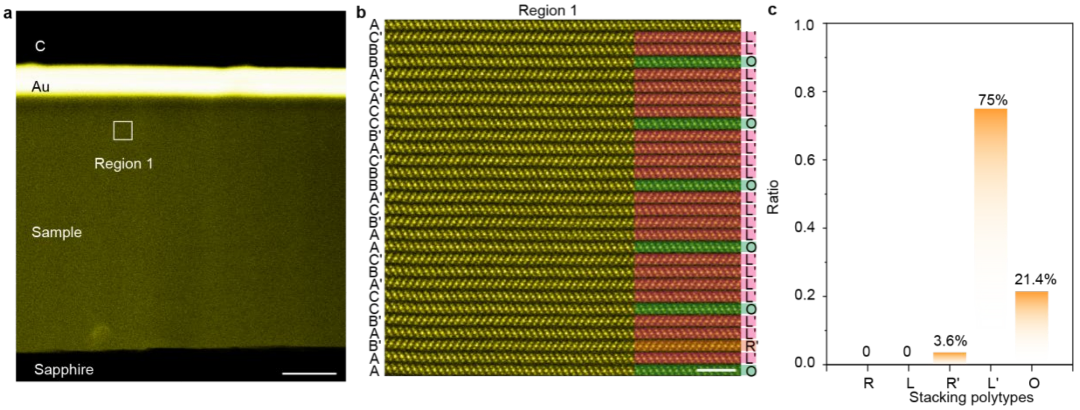

Supplementary Fig. 25. The ADF-STEM image of $SnSe_2$ (thickness = 474 nm) on a sapphire substrate. (a) A low-magnification ADF-STEM image showing atomic configuration of $SnSe_2$ slabs. (b) $SnSe_2$ crystals with mixed AA (O), AB' (R'), and AC' (L') stacking sequences. (c) Statistical analysis of the various stacking polytypes of $SnSe_2$ crystals grown on sapphire substrates. Scale bars: 100 nm in (a); 2 nm in (b).

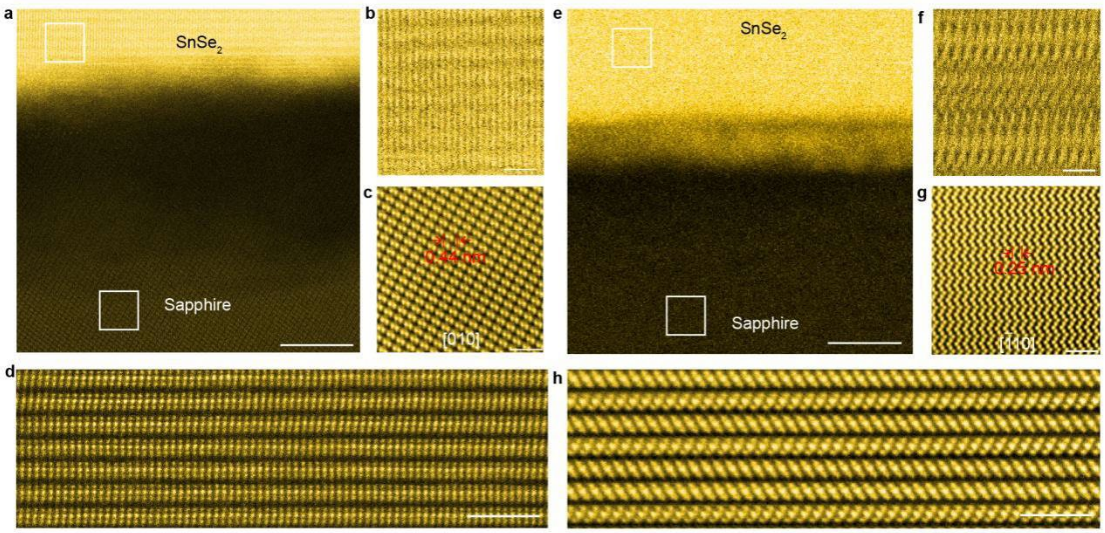

Supplementary Fig. 26. The atomic structure at the interface between $SnSe_2$ and sapphire substrate. (a) ADF-STEM image showing the atomic arrangement along the sapphire [010] zone axis. (b, c) Zoom-in ADF-STEM images providing $SnSe_2$ and sapphire substrate from **a**, respectively. (d) The arrangement of $SnSe_2$ crystals along the $[1\bar{1}0]$ Zone axis. Panels **b** and **d** indicate a lack of lattice matching between $SnSe_2$ and sapphire substrate. e, The ADF-STEM image along the $[\bar{1}10]$ zone axis of sapphire. (f, g) Zoom-in ADF-STEM images of $SnSe_2$ and Al_2O_3 crystals. h, Atomic structure of $SnSe_2$ along [100] zone axis. Scale bars: 5 nm in (a, e), 1 nm in (b, c), 10 nm in (d, h), 2 nm in (f, g).

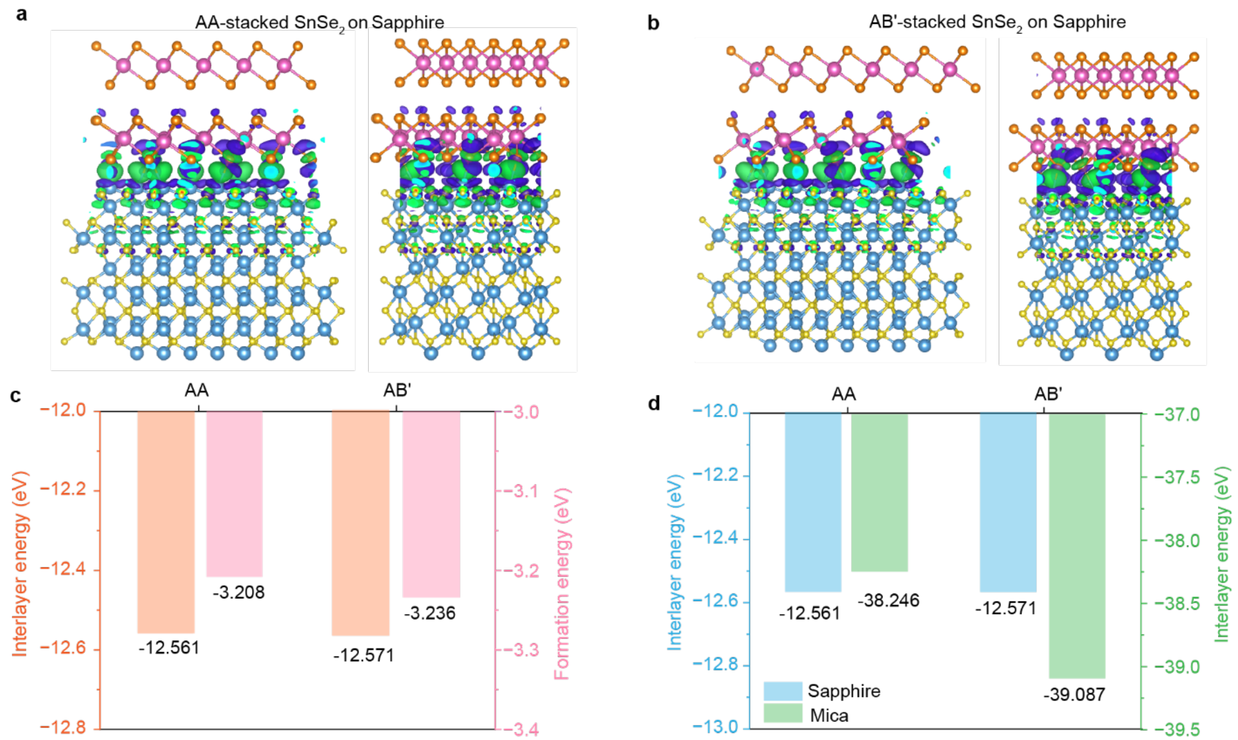

Supplementary Fig. 27. DFT calculations of the interlayer energy and the formation energy for different stacking structures. (a, b) The simulated ADF-STEM images derived from the DFT-optimized AA- and AB'-stacked $SnSe_2$ on the sapphire surface. The right panel illustrates the differential charge density of both AA-, and AB'-stacked $SnSe_2$ on the sapphire substrate. The area in green is the region that gained electrons, and the area in purple represent those lost electrons. (c) The interlayer energy difference and the formation energy difference between AA and AB'-stacked $SnSe_2$ on the sapphire substrate. (d) The interlayer energy of AA and AB'-stacked $SnSe_2$ on sapphire and mica substrate, respectively.

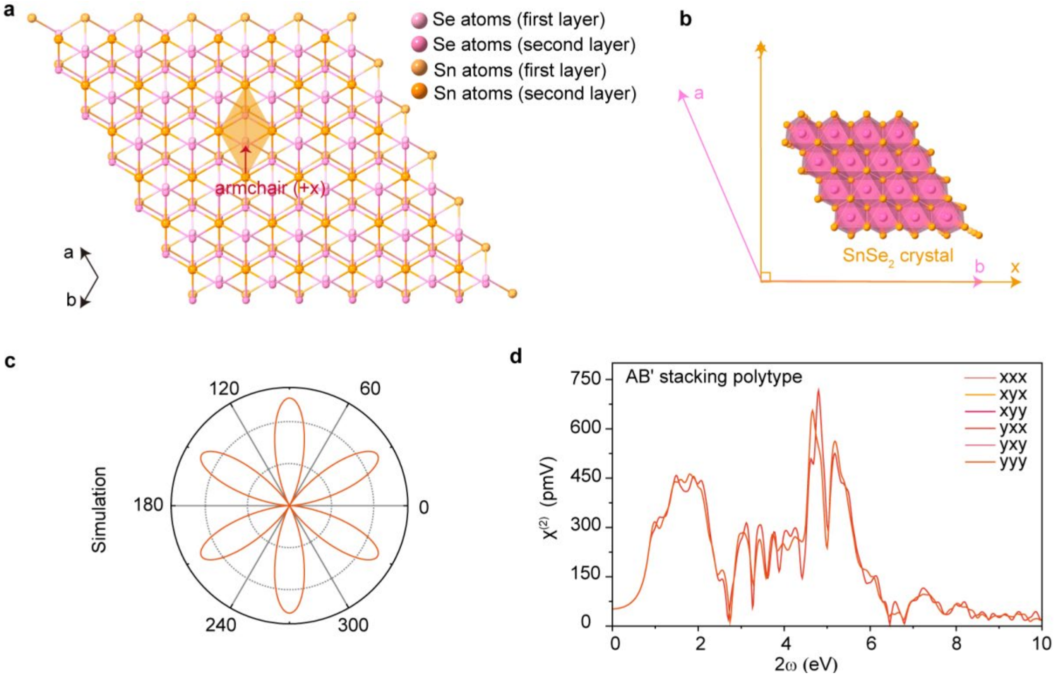

Supplementary Fig. 28. Calculated SHG response of AB'-stacked $SnSe_2$ crystals. (a) The schematic diagram illustrating the gliding direction of the AB'-stacked $SnSe_2$. The arrows correspond to the gliding direction along the armchair (+*x*) direction. (b) The transformation from crystal coordinates (lattice constant *a* and *b*) to SHG calculated coordinates (*x*, *y*). (c) Simulated polar plot pattern with six petals derived from AB'-stacked $SnSe_2$. (d) The calculated $\chi^{(2)}$ tensor elements of the AB'-stacked $SnSe_2$ with three nonzero tensors.

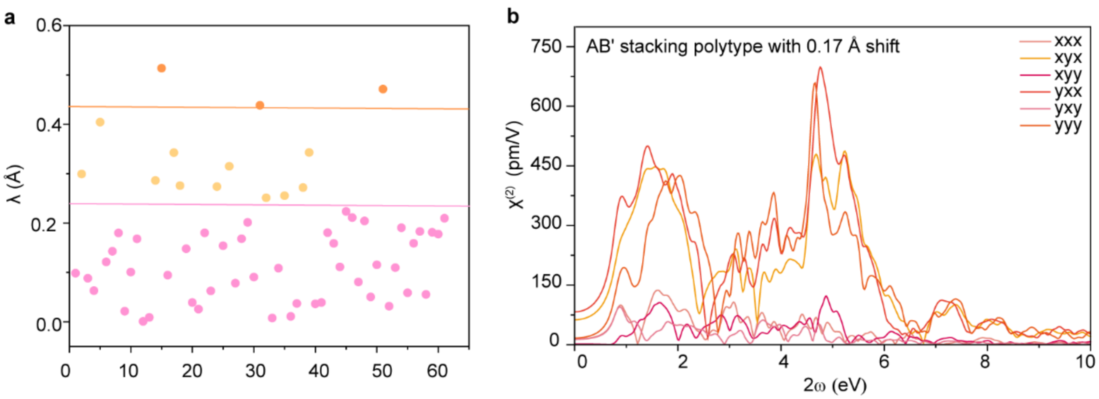

Supplementary Fig. 29. The displacements of Sn atoms. (a) The statistical result indicating a high possibility of Sn atomic displacements less than 0.22 Å based on ADF-STEM images (Fig. 4d) revealing the AB' stacking order. (b) The calculated $\chi^{(2)}$ tensor elements based on the AB'-stacked $SnSe_2$ with 0.17 Å displacement.

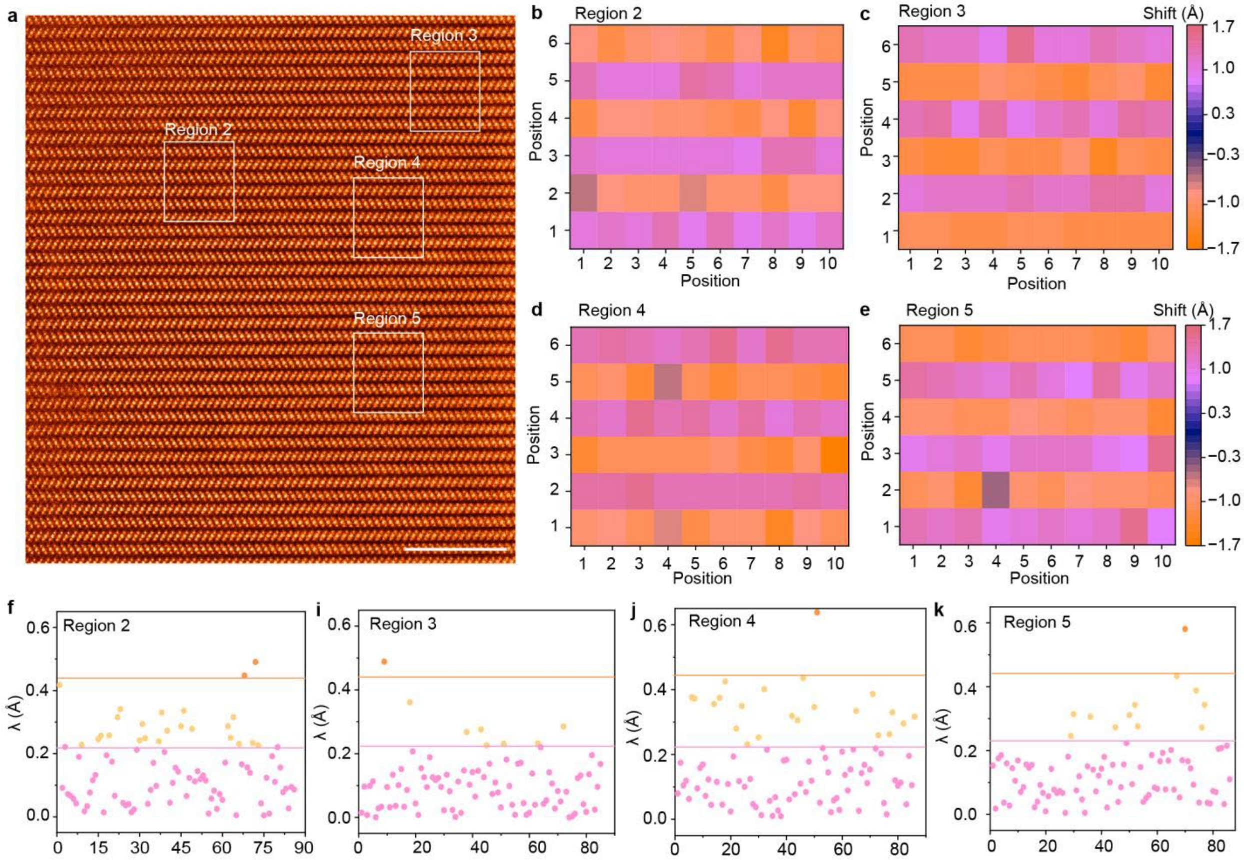

Supplementary Fig. 30. Statistical displacements analysis of Sn atoms in AB'-stacked $SnSe_2$. (a) The cross-sectional ADF-STEM image of AB'-stacked $SnSe_2$ with minor shifts. (b-e) Calculated Sn atom displacement maps from the white box regions 1-4 in a, respectively. (f-k) Statistical plots of the magnitude of Sn atom displacements in (f-k). The pixel size in (a) is 0.17 Å. Scale bar: 5 nm in (a).

Pink dots represent the displacement values less than 0.22 Å. The yellow region suggests the values are between 0.22 Å and 0.44 Å. Orange dots represent the shift values larger than 0.44 Å. From the four panels, we can infer that the atomic displacement is mainly less than 0.22 Å, consistent with the calculated slipping energy. ADF-STEM images combined with DFT calculations demonstrate that the minor displacements of a layer with respect to adjacent layers can spontaneously occur, once the energy barrier can be overcome at the given temperature.

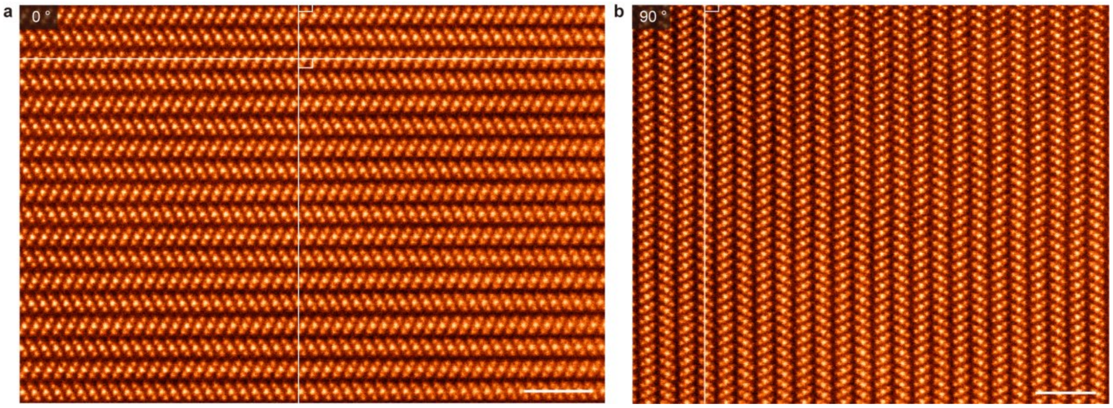

Supplementary Fig. 31. ADF-STEM images of the same $SnSe_2$ area with different scanning angles, e.g., 0° (a) and 90° (b). Scale bars: 2 nm. Interlayer gliding can be observed when the scanning angle is 0°, but they are absent when the scanning angle is 90°. These results confirm that the experimentally observed interlayer gliding is irrelevant to the sample drift during the image acquisition.

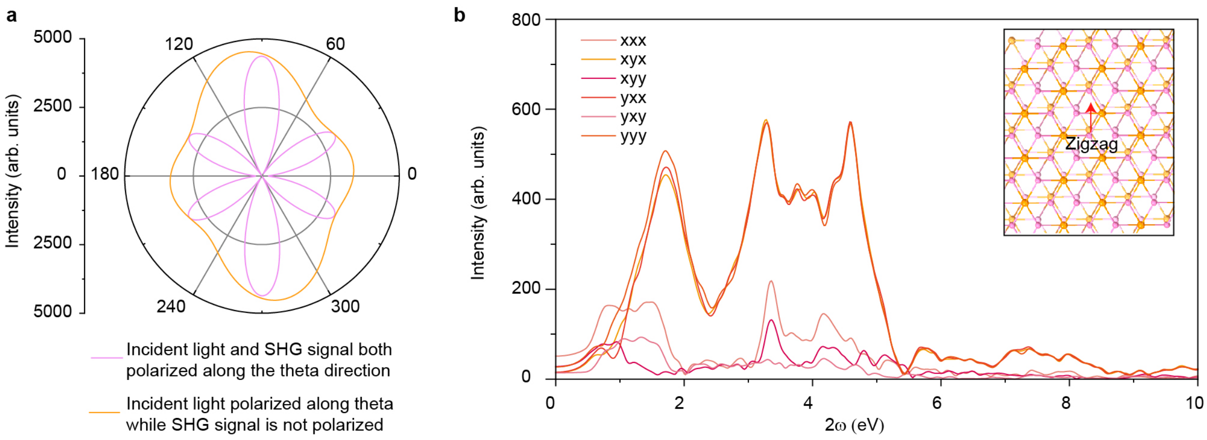

Supplementary Fig. 32. The calculated SHG results of AB'-stacked $SnSe_2$ with minor gliding along zigzag direction. (a) The calculated azimuthal SHG polarization dependence with a 1064 nm laser. The detection beams are polarized (pink) and not polarized (orange). (b) The $\chi^{(2)}$ tensor elements of the AB'-stacked $SnSe_2$ with 0.17 Å displacement along the zigzag direction. An atomic model with interlayer gliding along the zigzag gliding was depicted inset.

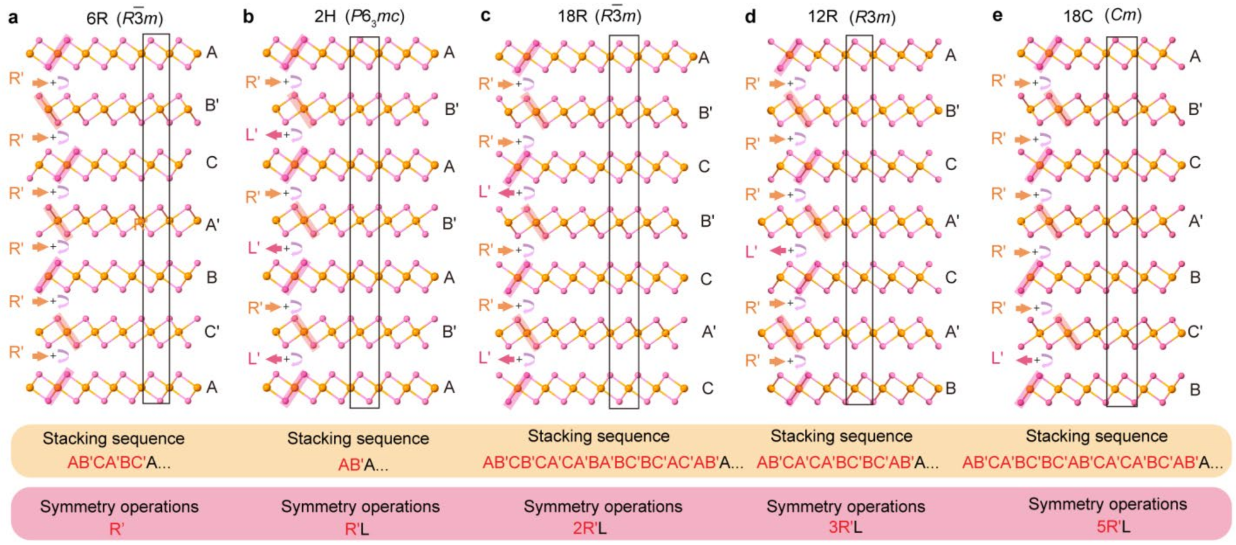

Supplementary Fig. 33. The atomic models of five stacking polytypes in $SnSe_2$ induced by a series of interlayer symmetry operations. Atomic structures of $SnSe_2$ displaying 6R ($R\bar{3}m$) (a), 2H ($P6_3mc$) (b), 18R ($R\bar{3}m$) (c), 12R (R3m) (d), and 18C (Cm) (e), space group. The in-plane displacement operation is represented as an orange arrow and the interlayer inversion operation is labelled by a pink arrow. The Sn and Se atoms are highlighted in orange and pink, respectively.

Firstly, all structures feature interlayer gliding and interlayer mirror symmetry operations between two adjacent $SnSe_2$ layers, and the major symmetry operations come from the crystallographic direction of the interlayer gliding. As illustrated in Fig. 5a-b, the interlayer gliding along the [120] direction from A to B is defined as an R operation, while the gliding along $[\bar{1}20]$ direction from A to C is termed as L. The operation in AB' stacking structure, in which the first $SnSe_2$ slabs glide against the bottom layer as well as rotating 180 ° was defined as R'. And the glide along $[\bar{1}20]$ direction with inversion symmetry is regarded as L'.

As for the 6R stacking registry, the bottom layer of two adjacent layers always translates $\frac{1}{\sqrt{3}}a$ along the [120] direction relative to the upper layer, resulting in an AB'CA'BC' sequence with a periodic unit containing six layers (Supplementary Fig. 33a). Besides, another stacking sequence namely 2R'L' is

observed with a periodicity of 18 layers, featuring a much lower symmetry structure, which belongs to $R\bar{3}m$ (Supplementary Fig. 33c). Significantly, 3R'L' and 5R'L' stacking landscapes, with a unit cell of 12 layers and 18 layers respectively (Supplementary Fig. 33d and e), also be observed. The corresponding space groups are labeled as R3m and Cm, respectively. The stacking polytypes of 12R exhibit broken centrosymmetric character, demonstrating the relationship between the crystal symmetry and interlayer behaviors.

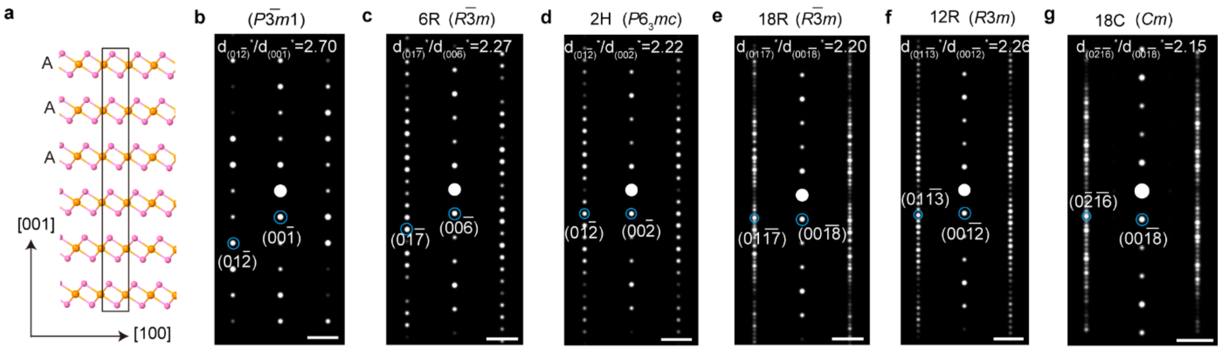

Supplementary Fig. 34. The diffraction analysis of the as-grown $SnSe_2$ with an atlas of stacking polytypes. (a) Atomic model of AA-stacked $SnSe_2$ along [010] zone axis. The simulated electron diffraction patterns of $P\bar{3}m1$ (b), 6R (c), 2H (d), 18R (e), 12R (f) and 18C (g) along the [100] direction. Scale bars: 2 nm^{-1}.

The interlayer gliding disrupts the crystal symmetry of $SnSe_2$ crystals, thus affecting the periodicity and intensity of diffraction patterns. Compared with standard AA-stacked $SnSe_2$, the FFT patterns of AB'CA'BC'A-stacked $SnSe_2$ with $R\bar{3}m$ space group reveals an additional set of diffusive Bragg spots suggesting long-range ordering in AB'CA'BC'A-stacked $SnSe_2$ (Supplementary Fig. 34b-g).

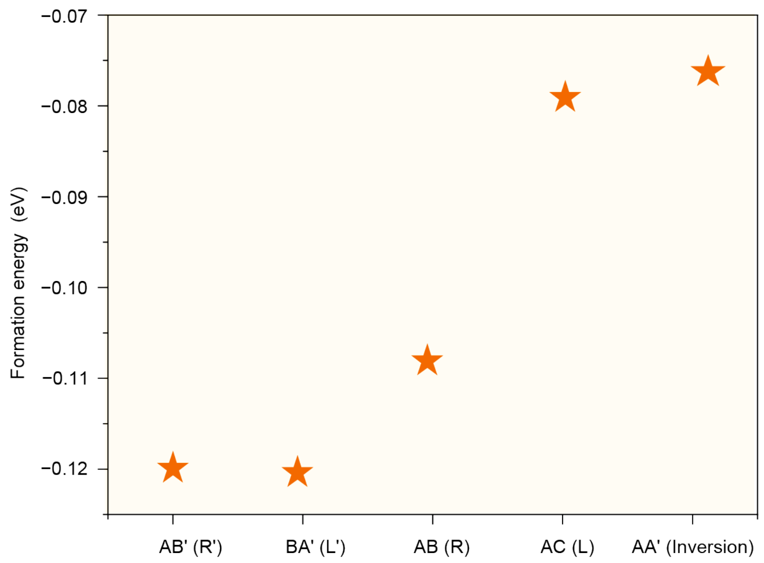

Supplementary Fig. 35. The calculated formation energy of the five symmetric operations without the consideration of mica substrate.

The energy of AB' and BA' stacking sequences are thermodynamically equivalent and represent the most stable stacking polytypes.

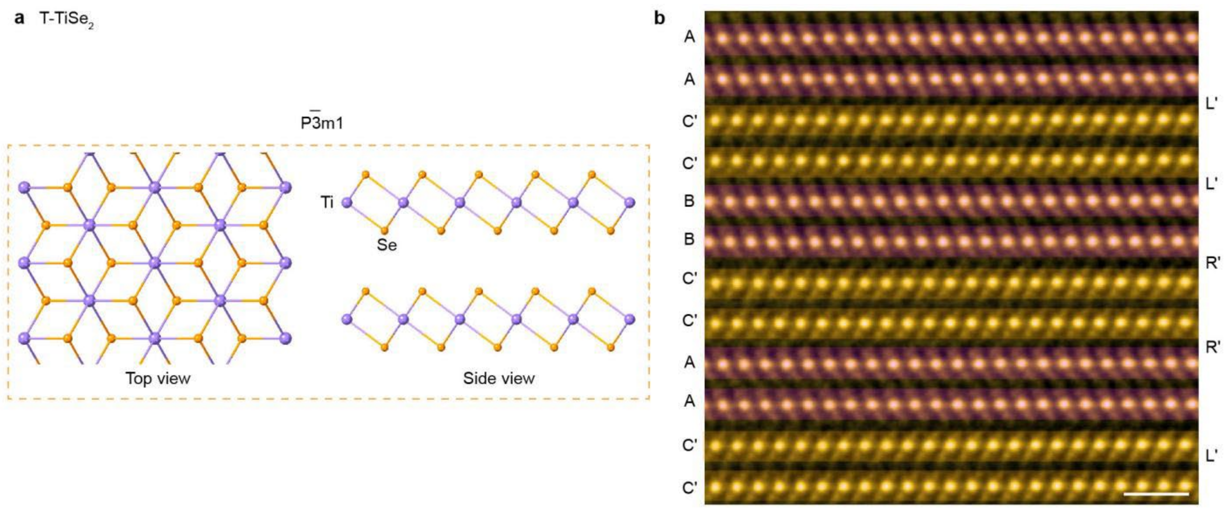

Supplementary Fig. 36. Cross-sectional analysis of CVD-grown $TiSe_2$ crystals. (a) Atomic model of the intrinsic AA-stacked $TiSe_2$ from the top (left panel) and side view (right panel). (b) ADF-STEM image of T-phase $TiSe_2$ with an AAC'C'BBC'C' stacking sequence. Scale bar: 1 nm.

2. Supplementary Table 1-10

Stacking orders	E (eV)*	E (eV/f.u.)	ΔE (meV/f. u.)	Formation energy (eV/f.u.)
AB'/BC'/CA'	-24.773	-12.386	1	-0.121
AC'/BA'/CB'	-24.773	-12.386	1	-0.121
AA/BB/CC	-24.774	-12.387	0	-0.122
AB/BC/CA	-24.746	-12.373	14	-0.108
AC/BA/CB	-24.689	-12.344	43	-0.079
AA'/BB'/CC'	-24.749	-12.375	11.5	-0.110

* Total energy E (eV); f. u.= formula unit

Supplementary Table 1. The DFT calculated formation energies of $SnSe_2$ as a function of stacking order without accounting for the substrate effect.

Work function (eV)	AA stacking	AB' stacking
Mica	3.049	3.0512
$SnSe_2$	5.888	5.9149

Supplementary Table 2. DFT results showing work functions of mica crystal, AA- and AB'-stacked $SnSe_2$ crystals with the mica/$SnSe_2$ system.

	Total energy (eV)	Substrate energy (eV)	$SnSe_2$ energy (eV)	Interlayer energy (eV)
AA-stacked $SnSe_2$/Sapphire	-2212.726	-1903.664	-296.501	-12.561
AB'-stacked $SnSe_2$/Sapphire	-2213.862	-1904.770	-296.521	-12.571
AA-stacked $SnSe_2$/mica	-3283.314	-2457.692	-787.375	-38.246
AB'-stacked $SnSe_2$/mica	-3284.557	-2458.615	-786.855	-39.087

Supplementary Table 3. The interlayer energy of AA- and AB'-stacked $SnSe_2$ on sapphire and mica substrate.

Work function (eV)	AA stacking	AB' stacking
Sapphire	6.040	6.154
$SnSe_2$	5.606	5.921

Supplementary Table 4. Work functions of sapphire, AA-stacked and AB'-stacked $SnSn_2$ crystals in sapphire/$SnSe_2$ system.

	Formation energy (eV)
AA-stacked $SnSe_2$/Sapphire	-3.208
AB'-stacked $SnSe_2$/Sapphire	-3.236
AA-stacked $SnSe_2$/Mica	-7.949
AB'-stacked $SnSe_2$/Mica	-8.064

Supplementary Table 5. The formation energy of AA- and AB'-stacked $SnSe_2$ on sapphire and mica substrate.

	Average (Å)	Maximum (Å)	Minimum (Å)	0.22 ≤λ (Å)≤ 0.44	Λ (Å)<0.44
Region 1	0.173	0.554	0.001	26.3%	4.9%
Region 2	0.145	0.451	0.001	16.3%	1.2%
Region 3	0.118	0.449	0.000	16.3%	1.2%
Region 4	0.184	0.678	0.003	16.3%	3.5%
Region 5	0.132	0.540	0.000	16.3%	1.2%
Simulation	Error: 0.0095%				

Supplementary Table 6. Calculated gliding magnitude λ based on ADF-STEM images (Fig. 4d and Supplementary Fig. 30b-e).

More than half of the atoms display displacements smaller than 0.22 Å, and a few atomic displacements reach 0.68 Å, which matches well with the calculation of interlayer slipping energy.

Slip distance (Å)	E (eV)	ΔE (meV/f. u.)
0 (AC')	-24.766	0.000
0.22	-24.763	1.543
0.44	-24.757	4.493
0.66	-24.750	7.840
0.88	-24.745	10.348
1.10 (AA')	-24.749	8.721
1.32	-24.745	10.376
1.54	-24.750	7.939
1.76	-24.757	4.768
1.92	-24.763	1.614
2.20 (AB')	-24.766	0.009

Supplementary Table 7. DFT calculated the gliding barrier of AC'-stacked $SnSe_2$ gliding along the [120] for 2.20 Å distances.

	Total energy (eV)	Interlayer energy (eV)	Formation energy (eV)
BA'-stacked $SnSe_2$/mica (L')	-3283.496	-38.486	-7.708
AB'-stacked $SnSe_2$/mica (R')	-3284.557	-39.087	-8.604

Supplementary Table 8. The interlayer energy as well as formation energy between R' and L' operations after taking into account of mica substrate.

Stacking sequence	SnI_2 temperature	Gas flow (Ar)	Gas flow (H_2)
AA stacked $SnSe_2$	320 ℃	20 sccm	0.4 sccm
AB' (2H) stacked $SnSe_2$	340 ℃	20 sccm	0.4 sccm
6R stacked $SnSe_2$	340 ℃	40 ~ 80 sccm	0.8 sccm
18R stacked $SnSe_2$	340 ℃	40 ~ 80 sccm	0.8 sccm
12R stacked $SnSe_2$	340 ℃	40 ~ 80 sccm	0.8 sccm
18C stacked $SnSe_2$	340 ℃	40 ~ 80 sccm	0.8 sccm

Supplementary Table 9. The detailed growth conditions of $SnSe_2$ crystals with different stacking sequences.

	Symmetry operations	$\chi^{(2)}_{xxx}$	$\chi^{(2)}_{xyx}$	$\chi^{(2)}_{xyy}$	$\chi^{(2)}_{yxx}$	$\chi^{(2)}_{yxy}$	$\chi^{(2)}_{yyy}$
6R ($R\bar{3}m$)	R'	0.057	0.113	0.016	0.065	0.026	0.247
2H ($P6_3mc$)	R'L'	0.003	242.471	9.70×10^{-4}	242.586	0.003	244.358
18R ($R\bar{3}m$)	2R'L'	0.002	0.035	0.002	0.046	0.002	0.110
12R (R3m)	3R'L'	0.003	61.809	0.001	61.743	0.002	61.566

Supplementary Table 10. Calculated six components $\chi^{(2)}$ of various stacking polytypes for T-phase $SnSe_2$. The $SnSe_2$ crystals with $P6_3mc$ and R3m polytypes exhibit nonzero SHG response with an exciting light at 1064 nm.

3. Supplementary Note. 1-6

Supplementary Note 1: DFT calculations

DFT calculations were performed using the generalized gradient approximation for the exchange-correlation potential, the projector augmented wave method[1,2], and a plane-wave basis set as implemented in the Vienna ab initio simulation package[3]. The Perdew-Burke-Ernzerhof functional were considered at the DFT-D3 level[3].

For the calculation of AA and AB'-stacked $SnSe_2$ without considering the existence of substrate, we employed a kinetic energy cut-off of 600 eV for the plane-wave basis set to perform geometric and interlayer energy calculations. A k-mesh of 21 × 21 × 1 was adopted to sample the first Brillouin zone. All atoms were relaxed until the residual force per atom reached 5×10^{-3} eV $Å^{-1}$ and the energy convergence criteria were set to 1×10^{-5} eV.

Considering the effect of substrate on the interlayer energy of AA and AB'-stacked $SnSe_2$ crystals, the calculation was performed using a kinetic energy cut-off of 450 eV for the plane-wave basis set. The k-mesh was set to 9 x 5 x 5 to sample the first Brillouin zone. In this case, the relaxation of all atoms in the unit cell was allowed until the residual force per atom reached 2×10^{-2} eV $Å^{-1}$ and the energy convergence criteria was 1×10^{-5} eV. Notably, the supercells used in this calculation were $4 \times 4\sqrt{3}$ $SnSe_2$ and 3×3 mica, with a mismatch of 4 % tensile strain for mica. Additionally, $2\sqrt{3} \times 2\sqrt{3}$ $SnSe_2$ with 3×3 sapphire, resulting in a mismatch of 8 % compressive strain for sapphire.

Supplementary Note 2: The calculation results of $SnSe_2$ crystals on sapphire substrate.

The typical c-plane oriented sapphire generally exhibits an aluminium (Al) surface termination, as depicted in Supplementary Fig. 23. Experimentally, $SnSe_2$ crystals did not exhibit perfect epitaxial alignment with the sapphire substrate under the same growth conditions (Supplementary Fig. 22). Specifically, ADF-STEM imaging revealed a significant off-axis deviation of sapphire substrate when $SnSe_2$ was aligned along its principal axis (Supplementary Fig. 26). For $SnSe_2$ crystals with a thickness of 474 nm, we qualified the stacking behaviors, finding that 75% exhibited BA' (L') stacking behaviors, 3.6 % with AB' (R') stacking orders and 21.4 % had intrinsic stacking slabs (Labelled as O).

Bader charge analysis further revealed that the charge transfer between the sapphire substrate and $SnSe_2$ was significantly weakened compared to that observed with mica (2.03 e^- for AA stacked $SnSe_2$ and 2.02 e^- for AB'-stacked $SnSe_2$). Furthermore, the work function of sapphire is approximately 6.0 eV, closely matching that of $SnSe_2$, whereas the variation in formation energy across different substrates can reach up to 4.0 eV, underscoring the critical influence of substrate choice in the material growth process (Supplementary Table 4).

Supplementary Note 3: ADF-STEM image analysis details

Quantitatively, we calculated the interlayer shifts (λ) using a customized Python script. The λ is defined as the horizontal distance between adjacent Sn atoms in neighboring layers. On the grounds of this, we found that the λ ranges from 0 to 0.55 Å. Meanwhile, there are no significant gliding preferences along the -x or +x directions (Supplementary Table 3). In parallel, the other four regions analyzed unexceptionally display comparable behaviors, highly indicating a disorder interlayer gliding manner for adjacent $SnSe_2$ slabs (Supplementary Fig. 17). The error of customized scripts, based on the simulated AB'-stacked $SnSe_2$, has been quantitatively measured, with a negligible value (0.0095%) (Fig. 4c). ADF-STEM images captured at different scan rotation angles of 0° and 90° eliminate the influence of image drift (Supplementary Fig. 18).

Supplementary Note 4: Strategy for the controlled synthesis of diverse $SnSe_2$ stacking registries with highly ordered superlattices.

During the synthesis of $SnSe_2$, we adjusted the Ar gas flow between 40 - 80 sccm to modulate the nucleation and growth dynamics, aiming to reconfigure $SnSe_2$ slabs. In principle, the gas flow rate should be suitable to give viscous laminar flow to ensure a homogeneous growth environment during the reaction process. However, there is always a velocity gradient of gases in the growth chambers, and decreases to zero when near the substrate, presenting a stagnant layer[4]. Choosing the high-rate gas flow is more likely to generate turbulence, which is concluded from the definition of the Reynolds coefficient. Meanwhile, the temperature gradient inside the furnace also accelerates the formation of turbulence. During the process, the collision of the atoms will be enhanced, which breaks the thermodynamic equilibrium and transforms the system into kinetic dominance[5,6]. The CVD growth process is generally complicated involving the sublimation and diffusion process of multiple precursors, maintaining the repeatability of 2D materials is still a big challenge[7]. Molecular beam epitaxy (MBE), owing to the high accuracy of controlling growth parameters, facilitate the precise manipulation of kinetic and thermodynamic behaviors to regulate the crystal symmetry in a variety of 2D materials[8].

Supplementary Note 5: SHG computational details

The first-principle calculations of SHG susceptibility in this work are performed within the Quantum ESPRESSO package[9,10]. Perdew, Becke, and Ernzerhof approximation for the exchange-correlation functional[11] is adopted and Van der Waals density functional corrections[12] is applied for atomic structure relaxations, where total energy changes less than 1 x 10^{-5} (a.u.) and all forces changes less than 1 x 10^{-4} (a.u.) between two consecutive iterations. Optimized norm-conserving Vanderbilt pseudopotentials[13,14] is used with an energy cutoff of 60 Ry. We choose 24 x 24 x 8 for Brillouin zone sampling in the self-consistent calculation of bulk AB'-stacked $SnSe_2$ (Supplementary Fig. 21a) and equivalent k-mesh for other structures.

For the nonlinear optical property calculations, we follow the independent particle approximation (IPA) formalism from Ref. [15]. The SHG susceptibility in the IPA is expressed as

$$\chi^{(2)}_{\alpha\beta\gamma}(-2\omega;\omega,\omega)=\frac{-ie^3}{\tilde{\omega}^3\hbar^2 m^3 V}\sum_{nml\mathbf{k}}\frac{p^{\alpha}_{nm\mathbf{k}}\{p^{\beta}_{ml\mathbf{k}},p^{\gamma}_{ln\mathbf{k}}\}}{\omega_{mn\mathbf{k}}-2\tilde{\omega}}\left[\frac{f_{nl\mathbf{k}}}{\omega_{ln\mathbf{k}}-\tilde{\omega}}+\frac{f_{ml\mathbf{k}}}{\omega_{ml\mathbf{k}}-\tilde{\omega}}\right]$$

where $\hbar\omega_{nm\mathbf{k}}=E_{n\mathbf{k}}-E_{m\mathbf{k}}$, $f_{nl\mathbf{k}}=f_{n\mathbf{k}}-f_{l\mathbf{k}}$, $p^{\alpha}_{nm\mathbf{k}}=\langle\psi_{n\mathbf{k}}|p^{\alpha}|\psi_{m\mathbf{k}}\rangle$, $\tilde{\omega}=\omega+i\eta$. $f_{n\mathbf{k}}$ is the occupation function. $E_{n\mathbf{k}}$ and $\psi_{n\mathbf{k}}$ are the single-particle energies and wave functions obtained from DFT calculation. $i\eta$ is a small imaginary part which originates from the adiabatic switching-on process in the perturbation theory for the derivation of $\chi^{(2)}_{\alpha\beta\gamma}$. In the practical calculations, we choose $\eta=0.05$ eV to represent the damping effect from the environment[16]. We use 36 x 36 x 12 k-mesh and include 26 valence and 26 conduction bands for the calculation of $\chi^{(2)}_{\alpha\beta\gamma}$ in bulk AB'-stacked $SnSe_2$ and parameters for other structures are chosen accordingly. For bilayer structure (Supplementary Fig. 21b), the cell volume *V* is overestimated due to the large artificial vacuum, so we renormalize $\chi^{(2)}_{\alpha\beta\gamma}$ using the bilayer thickness.

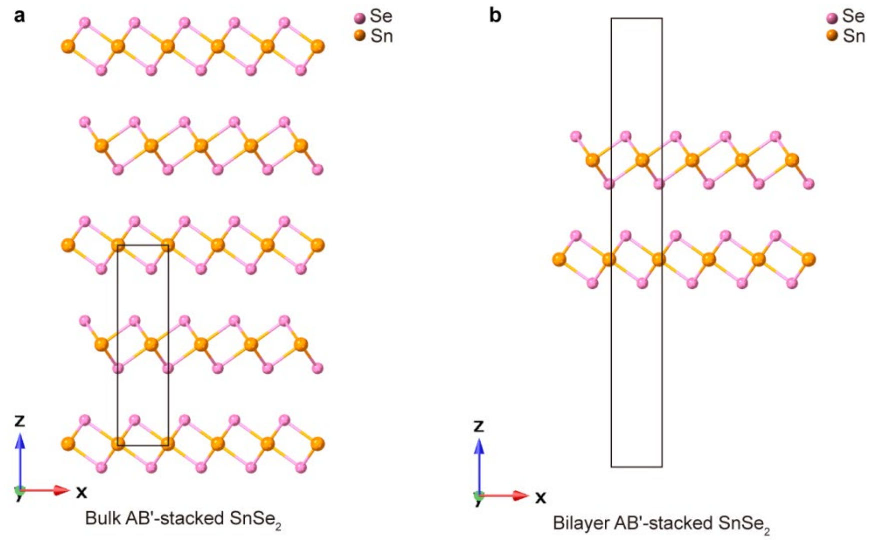

Supplementary Fig. 37. The atomic model chosen for SHG calculations. (a) Bulk AB'-stacked $SnSe_2$ structure. The black rectangle corresponds to the unit cell of AB'-stacked $SnSe_2$ crystals. (b) The model of bilayer $SnSe_2$ with the large artificial vacuum.

Supplementary Note 6: Polarization dependence of Raman spectra of the AB'-stacked $SnSe_2$

As shown in Supplementary Fig. 37b and c, the intensities of E_{2g} and A_{1g} mode are basically as circles no matter what directions were placed. To further validate the reliability of the experimental results, we performed a polarized Raman spectrum on $NbOCl_2$ crystal as a reference. Notably, the intensity of the 667 cm^{-1} peak exhibits a clear angular dependence, decreasing from a maximum at 0° to zero at 90°, and then gradually increasing from 90° to 180°.

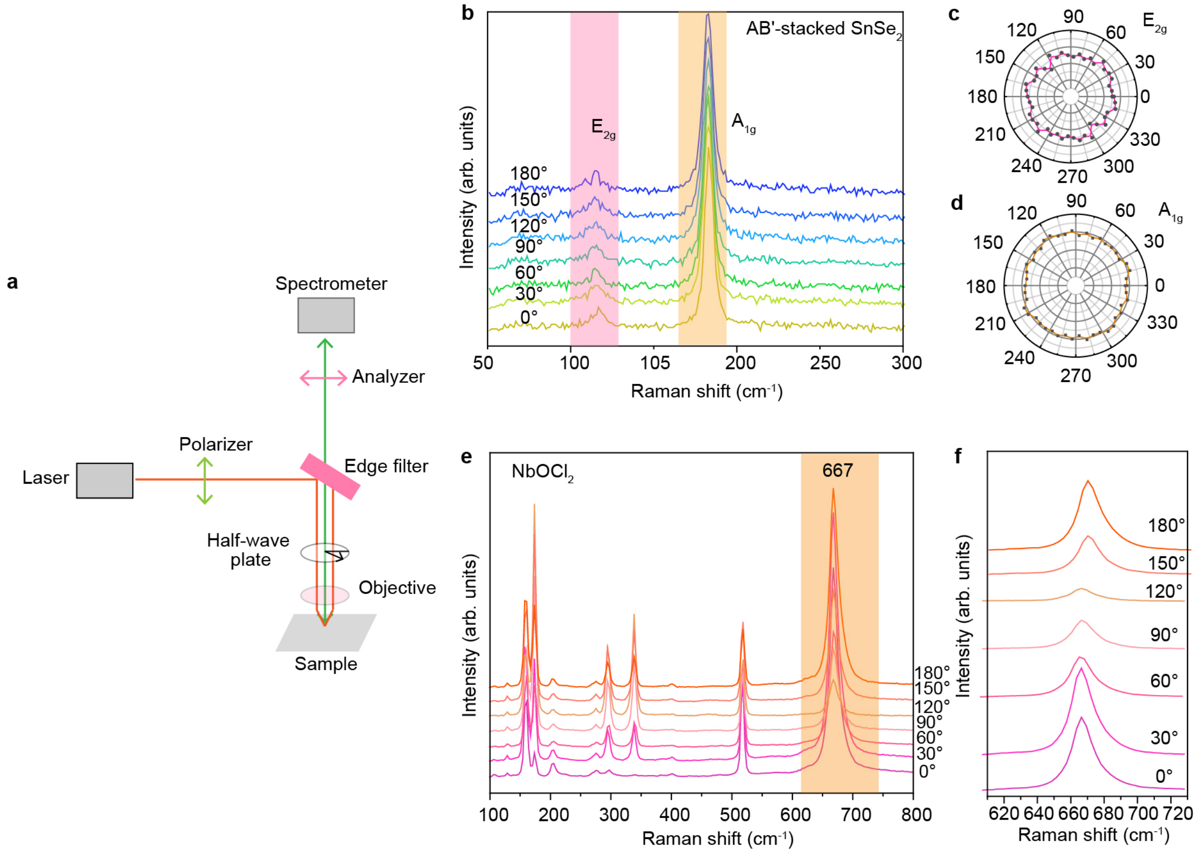

Supplementary Fig. 38. Polarization dependence of Raman spectra of the AB'-stacked $SnSe_2$ with the 532 nm excitation in parallel polarization configuration. (a) Schematic illustrations of polarized Raman measurements. (b) Angle-dependent polarized Raman intensity of AB'-stacked $SnSe_2$ crystal. Polar plot of Raman intensity for typical E_{2g} mode (c) and A_{1g} mode (d). (e) Raman spectra at various polarization angles under parallel configuration. (f) Polarization-dependent Raman intensity of 667 cm^{-1} in a $NbOCl_2$ crystal.

Supplementary References:

1. Blöchl, P. E. Projector augmented-wave method. *Physical Review B* **50**, 17953-17979 (1994).
2. Kresse, G. & Joubert, D. From ultrasoft pseudopotentials to the projector augmented-wave method. *Physical Review B* **59**, 1758-1775 (1999).
3. Kresse, G. & Furthmüller, J. Efficient iterative schemes for ab initio total-energy calculations using a plane-wave basis set. *Physical Review B* **54**, 11169-11186 (1996).
4. Bhaviripudi, S., Jia, X., Dresselhaus, M. S. & Kong, J. Role of kinetic factors in chemical vapor deposition synthesis of uniform large area graphene using copper catalyst. *Nano Lett.* **10**, 4128-4133 (2010).
5. Xu, M. *et al.* Reconfiguring nucleation for CVD growth of twisted bilayer MoS_2 with a wide range of twist angles. *Nat. Commun.* **15**, 562 (2024).
6. Zhang, Z. *et al.* Robust epitaxial growth of two-dimensional heterostructures, multiheterostructures, and superlattices. *Science* **357**, 788-792 (2017).
7. Tang, L., Tan, J., Nong, H., Liu, B. & Cheng, H.-M. Chemical vapor deposition growth of two-dimensional compound materials: Controllability, material quality, and growth mechanism. *Acc. Mater. Res.* **2**, 36-47 (2021).
8. Lee, J. H. *et al.* Dynamic layer rearrangement during growth of layered oxide films by molecular beam epitaxy. *Nat. Mater.* **13**, 879-883 (2014).
9. Giannozzi, P. *et al.* QUANTUM ESPRESSO: a modular and open-source software project for quantum simulations of materials. *J. Phys.: Condens. Matter* **21**, 395502 (2009).
10. Giannozzi, P. *et al.* Advanced capabilities for materials modelling with Quantum ESPRESSO. *J. Phys.: Condens. Matter* **29**, 465901 (2017).
11. Perdew, J. P., Burke, K. & Ernzerhof, M. Generalized Gradient Approximation Made Simple. *Phys. Rev. Lett.* **77**, 3865-3868 (1996).
12. Klimeš, J., Bowler, D. R. & Michaelides, A. Van der Waals density functionals applied to solids. *Physical Review B* **83**, 195131 (2011).
13. Hamann, D. R. Optimized norm-conserving Vanderbilt pseudopotentials. *Physical Review B* **88**, 085117 (2013).
14. Schlipf, M. & Gygi, F. Optimization algorithm for the generation of ONCV pseudopotentials. *Comput. Phys. Commun.* **196**, 36-44 (2015).

15 Leitsmann, R., Schmidt, W. G., Hahn, P. H. & Bechstedt, F. Second-harmonic polarizability including electron-hole attraction from band-structure theory. *Physical Review B* **71**, 195209 (2005).

16 Giuliani, G. & Vignale, G. *Quantum Theory of the Electron Liquid*. (Cambridge University Press, 2005).
